# Overexpression of *TCP9-like* gene enhances salt tolerance in transgenic soybean

**DOI:** 10.1371/journal.pone.0288985

**Published:** 2023-07-26

**Authors:** Zhuo Zhang, Yuanling Zhao, Yifan Chen, Yueming Li, Lijun Pan, Siyu Wang, Piwu Wang, Sujie Fan

**Affiliations:** 1 Plant Biotechnology Center, College of Agronomy, Jilin Agriculture University, Changchun, Jilin, People’s Republic of China; 2 Crop Resources Institute, Heilongjiang Academy of Agricultural Sciences, Harbin, Heilongjiang, People’s Republic of China; Central University of Haryana School of Life Sciences, INDIA

## Abstract

TEOSINTE BRANCHED1/CYCLOIDEA/PROLIFERATING CELL FACTOR (TCP) transcription factors are a plant-specific family and play roles in plant growth, development, and responses to biotic and abiotic stresses. However, little is known about the functions of the TCP transcription factors in the soybean cultivars with tolerance to salt stress. In this study, TCP9-like, a TCP transcription factor, was identified in the soybean cultivars exposed to salt stress. The expression of *TCP9-like* gene in the roots of salt-tolerant soybean cultivars was higher than that in salt-sensitive cultivars treated with NaCl. The overexpression of *TCP9-like* enhanced the salt tolerance of the salt-sensitive soybean cultivar ‘DN50’. In T2 generation, the plants with *TCP9-like* overexpression had significantly lower Na^+^ accumulation and higher K^+^ accumulation than the WT plants exposed to 200 or 250 mmol/L NaCl. The K^+^/Na^+^ ratio in the plants overexpressing *TCP9-like* was significantly higher than that in WT plants treated with 200 mmol/L NaCl. Meanwhile, the overexpression of *TCP9-like* up-regulated the expression levels of *GmNHX1*, *GmNHX3*, *GmSOS1*, *GmSOS2-like*, and *GmHKT1*, which were involved in the K^+^/Na^+^ homeostasis pathway. The findings indicated that *TCP9-like* mediated the regulation of both Na^+^ and K^+^ accumulation to improve the tolerance of soybean to salt stress.

## Introduction

TEOSINTE BRANCHED1/CYCLOIDEA/PROLIFERATING CELL FACTOR (TCP) transcription factors (TFs) are a plant-specific family [1]. The members of the TCP family have a highly conserved TCP domain, which consists of 59 amino acid residues [2]. The TCP domain is a non-canonical basic helix-loop-helix (bHLH) structure, playing a role in DNA binding [3]. According to the differences of TCP domain sequences, the TCP members in *Arabidopsis thaliana* are classified into classes I and II. Class I includes 13 proteins, and class II includes 11 proteins and is composed of the angiosperm-specific CYC/TB1 subclade and the ubiquitous CIN subclade [3, 4]. The TFs of TCP family are central regulators specifically binding to the *cis*-elements in the promoter of a target gene. The members of class I prefer to bind to the sequences with the motif of GGNCCCAC, while class II tends to bind to the motif of G(T/C)GGNCCC [2, 5–7].

TCPs play pivotal roles in a variety of life activities, such as seed germination, leaf morphogenesis, flower development, flavonoid biosynthesis, hormone signal transduction, effector-triggered immunity, and stress responses [8–16]. AtTCP14 and AtTCP15 mediate seed germination by regulating the production of gibberellins [11]. Wang et al. [17] reported that the tubers of the potato lines overexpressing StTCP15 sprouted in advance, while those of the lines with down-regulated StTCP15 expression showed delayed sprouting. The overexpression of *SlTCP26* promoted lateral branch development and suppressed the expression of the genes in indole-3-acetic acid (IAA) signaling [18]. Western blotting and yeast two-hybrid assay showed that the secreted AYWB protein SAP11_AYWB_ of phytoplasma specifically interacted with class II TCPs and destabilize TCPs in *Arabidopsis*, which then displayed axillary branching and leaf shape changes [19]. The overexpression of *BjuBRC1-1* (a TCP gene) in the *Arabidopsis brc1* mutant delayed the flowering [20]. Li and Zachgo [21] pointed out that AtTCP3 interacted with R2R3-MYBs to positively regulate the flavonoid production and negatively regulate the auxin response. In apple, the overexpression of *MdTCP46* diminished the sensitivity to abscisic acid (ABA) and the resistance to drought stress [22]. TCPs play a regulatory role in directly mediating the expression of *LOX2*, thereby affecting the biosynthesis of jasmonic acid [23]. The overexpression of *OsTCP19* in *Arabidopsis* enhanced the tolerance to both salt stress and water shortage [24]. Willig et al. [25] found that TCP9 modulated the root architectural plasticity in response to nematode infections via ROS-mediated processes. However, few studies have been conducted concerning the TCPs in soybean exposed to salt stress.

The studies about the salt tolerance mechanism of soybean mainly focus on the accumulation of SO_4_^2-^, CO_3_^2-^, HCO_3_^-^, Cl^-^, Mg^2+^, Ca^2+^, Na^+^, and K^+^ involved in intracellular ion homeostasis and ion-specific damage [26–28]. Na^+^ is the primary cause of ion-specific damage in a variety of plants [29]. The high K^+^/Na^+^ ratio is of importance for plants to maintain a low concentration of intracellular Na^+^ [30–32].

Our previous study has demonstrated by RNA sequencing that the expression of a *TCP* gene (Glyma.07G080300.1) was up-regulated in the salt-tolerant soybean cultivar ‘JN30’ under salt stress. However, the role of this *TCP* gene in the accumulation of intracellular ions in different genetic backgrounds remains to be characterized. In this study, we obtained the full-length sequence of this *TCP* gene from ‘JN30’. The sequence analysis showed that it shared the highest homology with AtTCP9 and was named as *TCP9-like*. To examine the behavior of *TCP9-like* gene in different genetic backgrounds, we employed *Agrobacterium*-mediated transformation to introduce the overexpression vector into the salt-sensitive soybean cultivar ‘DN50’ and produced three stable lines with overexpression of *TCP9-like*. In T2 generation, we evaluated the salt tolerance of the lines treated with 150, 200, and 250 mmol/L NaCl. Furthermore, we measured and compared the Na^+^ content, K^+^ content, and K^+^/Na^+^ ratio in *TCP9-like*-overexpressing and WT plants. Finally, we determined the expression levels of *GmNHX1*, *GmNHX3*, *GmSOS1*, *GmSOS2-like*, and *GmHKT1* involved in the K^+^/Na^+^ homeostasis pathway in *TCP9-like*-overexpressing and WT plants. The results suggested that *TCP9-like* improved the salt tolerance of soybean by regulating both Na^+^ and K^+^ accumulation.

## Materials and methods

### Plant materials and growth conditions

The salt-tolerant soybean cultivars ‘JN30’ and ‘JN18-2’ and the salt-sensitive soybean cultivars ‘DN50” and ‘JN18-7’ were used in this study. ‘JN30’ was used for gene isolation, and ‘DN50’ for soybean transformation. The seeds of the previous generation were sown in the pots filled with sterile vermiculite in a growth chamber with a 14-h photoperiod (at a light intensity of 350 mol m^-2^s^-1^) at 22°C /18°C (day/night) and relative humidity of 70% ± 10% at Jilin Agriculture University.

### RNA isolation and gene cloning

Trizol reagent (Invitrogen, China) was used to extract the total RNA from leaves of ‘JN30’ and a PrimeScript 1st Strand cDNA Synthesis Kit (TaKaRa, Dalian, China) to synthesize the first-strand cDNA. The sequence of soybean endogenous gene Glyma.07G080300.1 was downloaded from Phytozome (http://www.phytozome.net/). The full-length coding sequence (CDS) of Glyma.07G080300.1 was amplified from the cDNA with the primers *TCP9*-*F*/*R* (**S1 Table**). The PCR procedure: 94°C for 5 min, followed by 30 cycles of 94°C for 30 s, 58°C for 30 s, and 72°C for 2 min; final extension at 72°C for 10 min. The amplification products were cloned into pMD18-T (TaKaRa, Dalian, China) for sequencing.

### Phylogenetic analysis

To reveal the relationship of TCP-like in soybean with the TCPs in *Arabidopsis*, a phylogenetic tree was constructed with the full-length amino acid sequences. The neighbor-joining (NJ) tree was constructed in MEGA6.0 after multiple alignment of the sequences retrieved from Phytozome (http://www.phytozome.net/).

### qPCR

The One Step RT-PCR Kit (Code No. PCR-311, TOYOBO, Japan) was used for qPCR with the primers *TCP9*-qF/R and *GmEF1β*-qF/R in QuantStudio 3 (Thermo, United States) following the manufacturer’s protocol. The PCR protocol: 95°C for 1 min; 40 cycles of 95°C for 15 s, 60°C for 15 s, and 72°C for 45 s. The amplification product was confirmed by a melting curve established with one-degree intervals from 95°C to 60°C. *GmEF1β* was used as the reference gene. The primers of *TCP9-like*, *GmNHX1* (Glyma.10G158700.1), *GmNHX3* (Glyma.20G229900.1), *GmSOS1* (Glyma.08G092000.1), *GmSOS2-like* (Glyma.15G203700.1), *GmHKT1* (Glyma.06G271600.1), and *GmEF1β* (Glyma.17G001400.1) were designed by Primer Premier 5.0 (**S1 Table**). The expression levels of the target genes were calculated with the 2^−ΔΔCT^ method.

### Plasmid construction and soybean transformation

The full-length CDS of *TCP9-like* was amplified by RT-PCR with the primers *TCP9*-F/R (**S1 Table**) and inserted into pCAMBIA3301 with BAR as the selective marker. The construct 35S:*TCP9-like* was then transformed into *Agrobacterium tumefaciens* strain EHA105 via tri-parental mating. The cotyledonary nodes from the salt-sensitive soybean cultivar ‘DN50’ were used as explants for tissue culture and *Agrobacterium*-mediated transformation described by Guo et al. [33]. The transgenic soybean plants were verified by PAT/Bar LibertyLink strip (Envirologix, Portland, OR, USA) and qPCR. T2 transgenic soybean plants were selected for further phenotypic analysis.

### Salt tolerance assay

The 15-day-old (at the first-node stage (V1) [34]) plants were inoculated with 1/4 B5 nutrient solution supplied with 150, 200 or 250 mmol/L NaCl for 15 days. During salt stress treatment, leaves were harvested at the time points of 0, 6, 12, 24, and 48 h for RNA extraction and qPCR. When exposed to salt stress for 15 days, plant tissues were observed and photographed using a Nikon D700 camera. Meanwhile, the plants were harvested separately for the measurement of K^+^ and Na^+^ content by a flame photometer (410) [35, 36].

### Statistical analysis

All statistical methods are annotated in the figure captions. Three independent biological replicates were designed for each sample. The Student’s *t*-test at the levels of *P*<0.05 and *P*<0.01 were conducted for the numerical data.

## Results

### Gene structure and phylogenetic analysis relationship of TCP9-like

**Fig 1:**
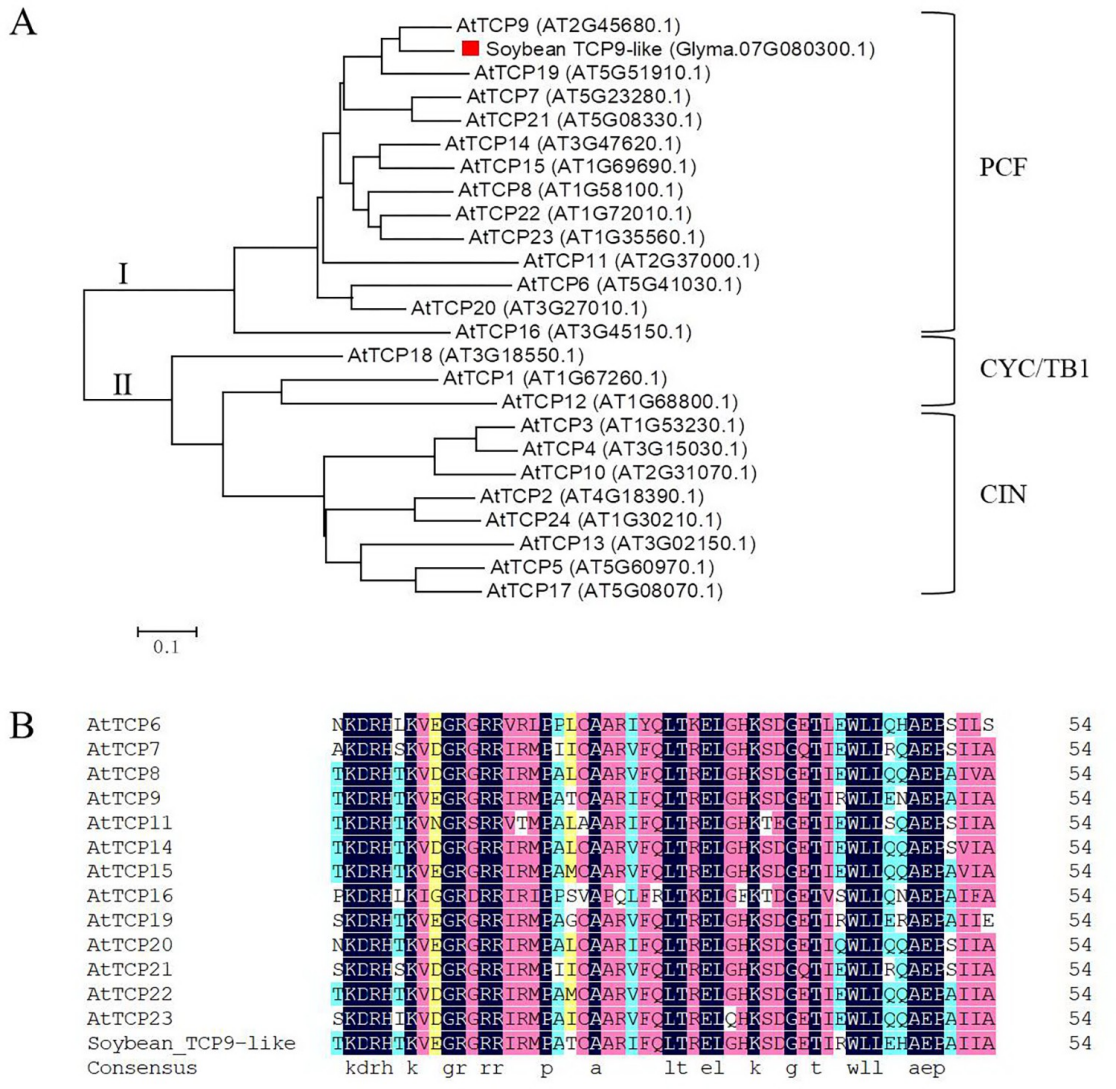
Phylogenetic tree and sequence alignment of TCP9-like in soybean. **(A)** The phylogenetic tree of TCP9-like in soybean and TCPs in *Arabidopsis*. A neighbor-joining (NJ) tree was constructed using in MEGA6.0 software after the multiple alignment with the bootstrap test of 1 000 replicates. The amino acid sequences of TCPs were downloaded from Phytozome (https://phytozome-next.jgi.doe.gov/). **(B)** The sequence alignment of the TCP domain of TCP9-like and the class Ⅰ TCPs from *Arabidopsis*.

The full-length nucleotide sequence of the *TCP* gene isolated from ‘JN30’ had an open reading frame (ORF) of 1 008 bp, encoding a protein composed of 336 residues and with a TCP domain at aa 71–125 (**S1 Fig**). To explore the evolutionary relationship between this TCP and the 24 TCPs from *Arabidopsis*, we constructed a phylogenetic tree using MEGA6.0 based on amino acid sequences. The result revealed that this TCP protein clustered in one branch with AtTCP9, belonging to the PCF subclass (class Ⅰ), and thus this soybean *TCP* gene was named as *TCP9-like* (**Fig 1A**). The TCP domain of TCP9-like and the class Ⅰ TCPs from *Arabidopsis* shared the amino acid sequence identity of 61.82%–98.18% and the TCP sequences were conserved across different species (**Fig 1B**).

### Expression pattern of *TCP9-like* in different soybean cultivars exposed to salt stress

**Fig 2:**
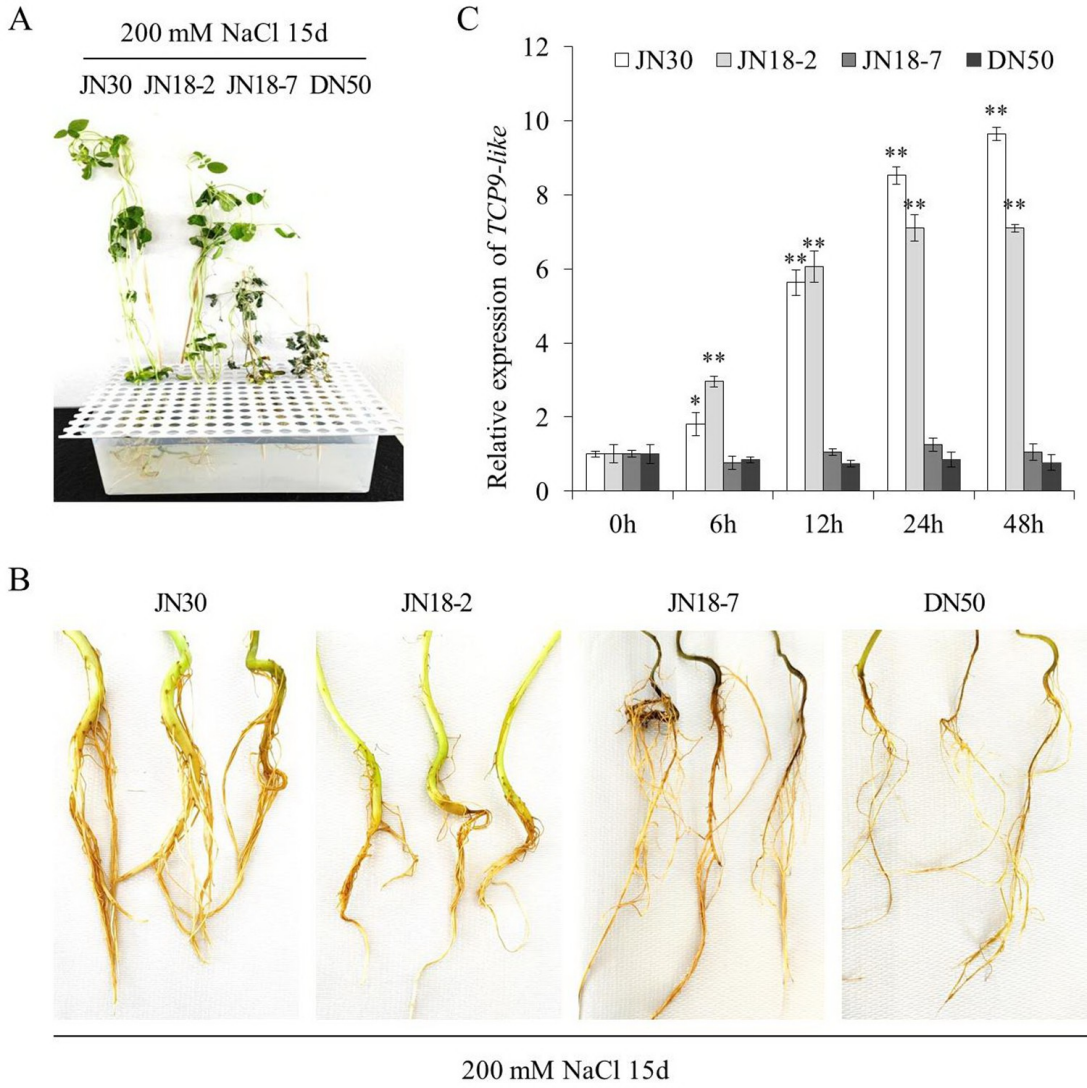
The expression pattern of *TCP9-like* gene in four soybean cultivars treated with 200 mmol/L NaCl. **(A)** Comparison of seedling performance of salt-tolerant and salt-sensitive soybean plants treated with NaCl for 15 days. **(B)** Comparison of root performance of salt-tolerant and salt-sensitive soybean plants treated with NaCl for 15 days. **(C)** The relative expression of *TCP9-like* in four soybean cultivars exposed to salt stress. Soybean root samples were collected 0 (control), 6, 12, 24, and 48 h after NaCl treatment. Three biological replicates were designed for each sample and the Student’s *t*-test (*P<0.05, **P<0.01) was performed to analyze the statistical significance of differences. Error bars represent ±SD.

The expression patterns of *TCP9-like* in the seedlings of four soybean cultivars were determined. After treatment with 200 mmol/L NaCl for 15 days, the expression of *TCP9-like* presented remarkable differences between the four soybean cultivars. As shown in **Fig 2A** and **2B**, salt-tolerant soybean cultivars (‘JN30’ and ‘JN18-2’) exhibited green leaves and normal roots, while salt-sensitive soybean cultivars (‘DN50’ and ‘JN18-7’) displayed yellow brown leaves and dead roots. The expression level of *TCP9-like* was higher in the two salt-tolerant soybean cultivars 12, 24, and 48 h after NaCl treatment (*p*<0.05), while that in two salt-sensitive soybean cultivars remained lower with no obvious changes during the treatment period (**Fig 2C**). The results suggested that *TCP9-like* may be involved in the response of soybean to NaCl stress.

### Overexpression of *TCP9-like* enhances salt tolerance of transgenic soybean

**Fig 3:**
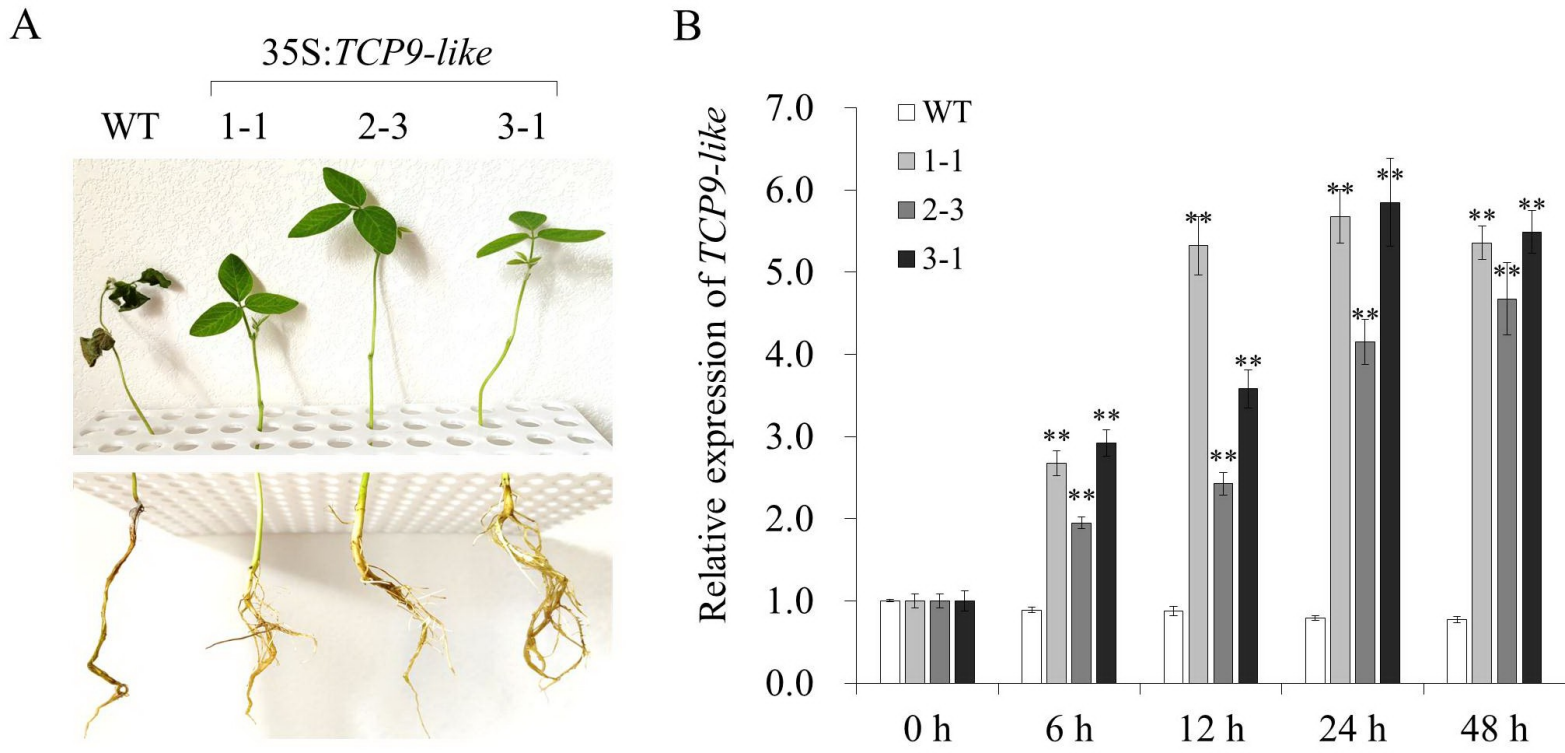
Salt tolerance of WT and *TCP9-like*-overexpressing soybean plants in T0 generation. **(A)** Phenotype of WT and *TCP9-like*-overexpressing soybean plants treated with 200 mmol/L NaCl for 15 days. WT, wild type. **(B)** The relative expression level of *TCP9-like* in transgenic soybean plants exposed to 200 mmol/L NaCl treatment for 0, 6, 12, 24, and 48 h. WT soybean plants were used as the control. Three biological replicates were designed for each sample and the Student’s *t*-test (*P<0.05, **P<0.01) was conducted to analyze the statistical significance of differences. Error bars represent ±SD.

To examine the behavior of *TCP9-like* in different genetic backgrounds, we employed *Agrobacterium*-mediated transformation to introduce the overexpression vector into the salt-sensitive soybean cultivar ‘DN50’. In T0 generation, three positive plants were examined for BAR protein by the LibertyLink strip (**S2 Fig**). In T1 generation, three positive transgenic plants of each line (1–1, 2–3, and 3–1) were randomly selected for qPCR and salt tolerance assay (**S3 Fig**). Under 200 mmol/L NaCl treatment, the transgenic plants overexpressing *TCP9-like* exhibited green leaves and strong stems, while the WT plants presented brown leaves and soft and wilting stems (**Fig 3A**). Meanwhile, the relative expression levels of *TCP9-like* in the three transgenic lines were much higher than that in WT plants (**Fig 3B**).

**Fig 4:**
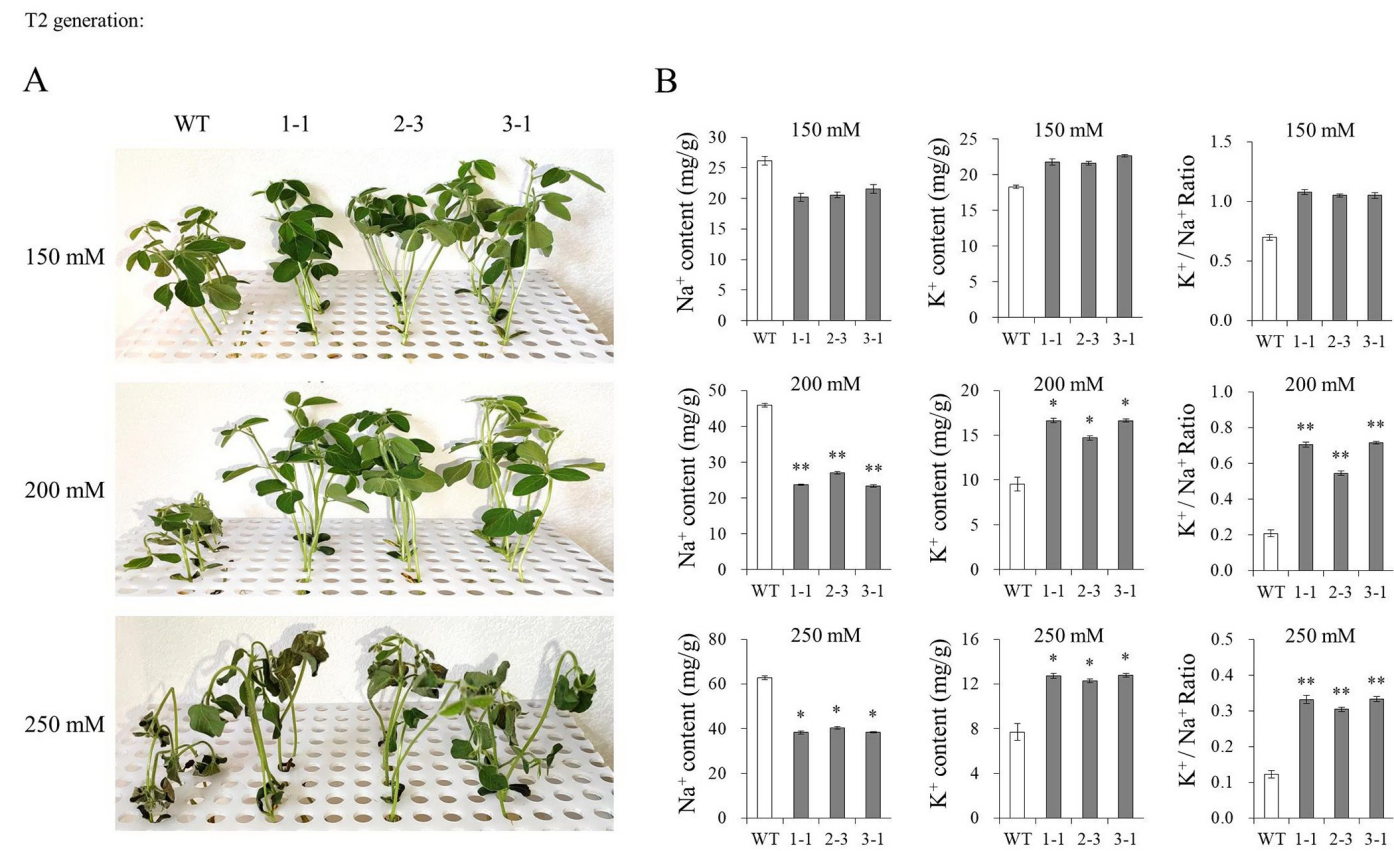
Salt tolerance of WT and *TCP9-like*-overexpressing soybean lines in T1 generation. **(A)** Salt tolerance of WT and three transgenic lines treated with 150, 200, and 250 mmol/L NaCl for 15 days. WT, wild type. **(B)** The Na^+^ content, K^+^ content, and K^+^/Na^+^ ratio of soybean plants treated with 150, 200, and 250 mmol/L NaCl for 15 days. WT, wild type soybean plants. Three biological replicates were designed for each sample and the Student’s *t*-test (*P<0.05, **P<0.01) was performed to analyze the statistical significance of differences. Error bars represent ±SD.

In T2 generation, three transgenic lines (1–1, 2–3, and 3–1) from each positive transgenic plant of T1 generation presented no segregation as revealed by the LibertyLink strip detection of BAR protein (**S4 Fig**). We evaluated the salt tolerance of three transgenic lines treated with 150, 200, and 250 mmol/L NaCl (**Fig 4A**). The WT plants treated with 200 or 250 mmol/L NaCl showed inhibited growth, as manifested by the curling stems and the shriveled browning leaves. However, the transgenic lines only showed such appearance under high salt stress (250 mmol/L NaCl). Furthermore, we measured the Na^+^ and K^+^ content in the three transgenic lines overexpressing *TCP9-like* (**Fig 4B**). Under 200 or 250 mmol/L NaCl treatment, the three transgenic lines had lower Na^+^ content (*P*<0.01 or *P*<0.05) and higher K^+^ content (*P*<0.05) than WT plants. The lower Na^+^ content and higher K^+^ content resulted in higher K^+^/Na^+^ ratios in the three transgenic lines than in WT plants (*P*<0.01). The results demonstrated that *TCP9-like* positively regulated salt tolerance of soybean under salt stress (200 or 250 mmol/L NaCl).

### Expression profiling of key genes involved in K^+^/Na^+^ homeostasis pathway

**Fig 5:**
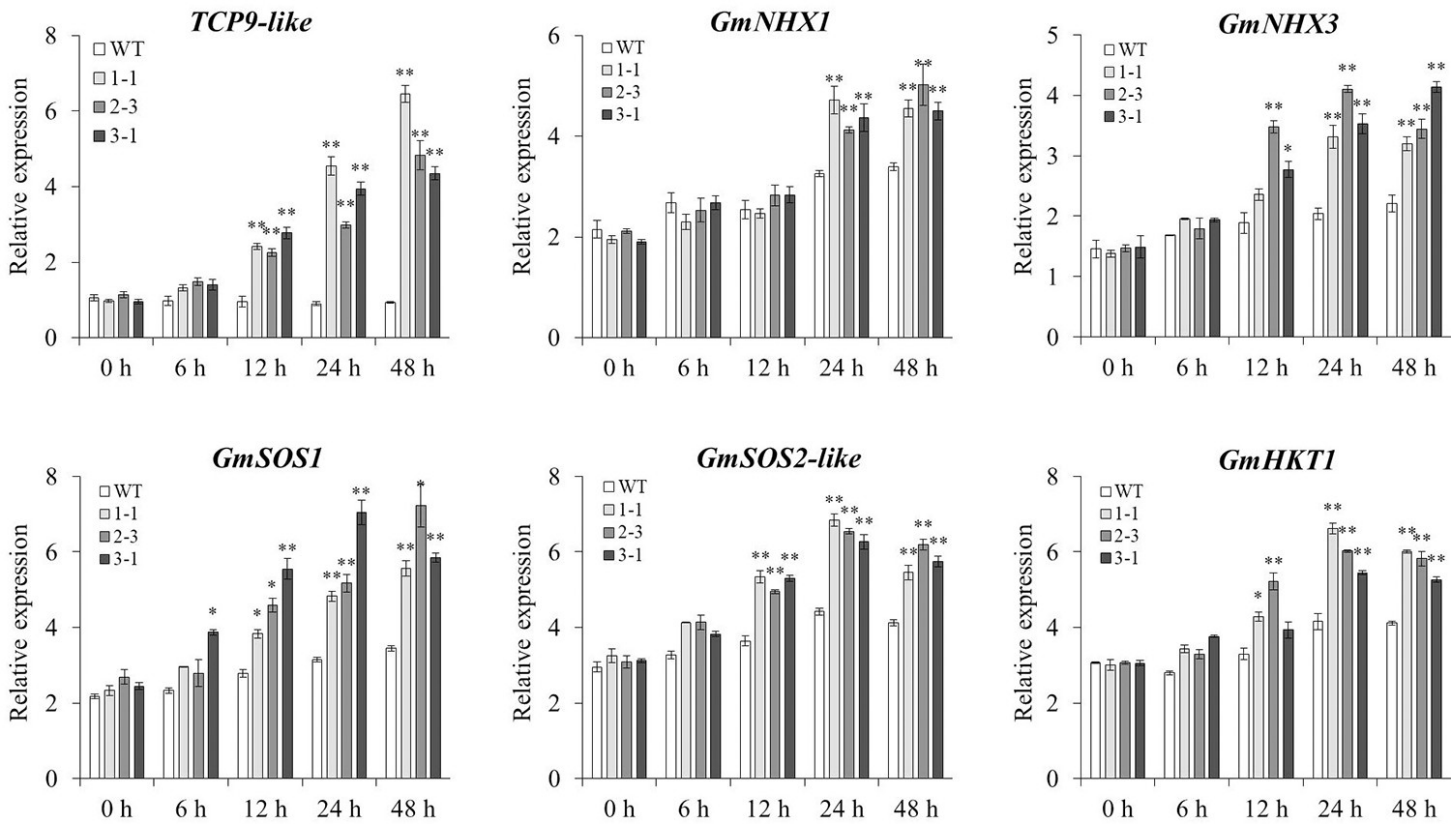
The expression levels of key genes involved in K^+^/Na^+^ homeostasis pathway in WT and three transgenic lines treated with 200 mmol/L NaCl. **WT, wild type.** Three biological replicates were designed for each sample and the Student’s *t*-test (*P<0.05, **P<0.01) was performed to analyze the statistical significance of differences. Error bars represent ±SD.

The expression levels of *GmNHX1*, *GmNHX3*, *GmSOS1*, *GmSOS2-like*, and *GmHKT1* involved in the K^+^/Na^+^ homeostasis pathway were measured in T2 generation of WT and three transgenic lines treated with 200 mmol/L NaCl. Because of the different genetic potential of genes in different genotypes, the increases in the expression levels of these genes were proportionally dissimilar for each genotype in response to salt stress. The expression levels of *GmNHX3*, *GmSOS1*, *GmSOS2-like*, and *GmHKT1* were in the three transgenic lines up-regulated (*P*<0.01 or *P*<0.05) compared with those of WT plants 12, 24, and 48 h after NaCl treatment. The expression level of *GmNHX1* in three transgenic lines was up-regulated (*P*<0.01) compared with that in WT plants 24 and 48 h after NaCl treatment (**Fig 5**).

## Discussion

TCPs comprise one of the plant-specific TF families and are ubiquitous in different plant species. Researchers have identified 25 TCPs in *Dendrobium catenatum* [37], 27 TCPs in *Cucumis sativus* [38], 66 TCPs in *Petunia axillaris* [39], 29 TCPs in *Zea mays* [40], 33 TCPs in *Populus euphratica* [41], 38 TCPs in *Gossypium raimondii* [42], and 6 TCPs in *Physcomitrella patens* [4]. The yield of soybean is affected by a variety of stress factors, especially salt stress. However, little information is available on the roles of the *TCP* genes in the response of soybean to salt stress. Here, a TCP transcription factor TCP9-like was cloned from soybean, which possessed a typical TCP domain and identified as a class I member (**S1 Fig** and **Fig 1**).

A number of *TCP* genes has been characterized and identified as key modulators of plant growth and development [38, 43]. AtTCP15 can directly regulate the expression of *GA20ox1*, *HBI1*, and *PRE6*, which participate in the gibberellin biosynthesis or plant growth and development, controlling the elongation of petiole and hypocotyl [44]. Using the yeast two-hybrid assay, Cao et al. [45] proved that the miR319 target gene *GhTCP4* interacted with GhHOX3 and functioned as a transcriptional repressor, thereby coordinating the fiber cell elongation and secondary cell wall biosynthesis. In addition, AtTCP15 can directly bind to the promoter of *SAUR63* to activate the gene expression, thus modulating gibberellin-dependent stamen filament elongation [46]. GrTCP11 was proved to be able to inhibit root hair elongation by down-regulating the jasmonic acid pathway in *A*. *thaliana*. Repression of the miR319 target gene *PvPCF5* (a *TCP* gene in switchgrass) improved salt tolerance by increasing ethylene synthesis and accumulation [16]. A new study indicated that overexpression of *PeTCP10* enhanced salt tolerance of transgenic *Arabidopsis* at the vegetative growth stage [47]. In this study, qPCR results showed that *TCP9-like* was highly expressed in salt-tolerant soybean cultivars after treatment with 200 mmol/L NaCl (**Fig 2**). The transgenic plants overexpressing *TCP9-like* exhibited much better phenotypes than WT plants under 200 mmol/L NaCl treatment for 15 days (**Fig 3**). These findings suggest that *TCP9-like* gene is a positive regulator for the response to salt stress.

Maintaining low Na^+^ and high K^+^ concentrations is an effective method for plants to deal with salt stress, and a high K^+^/Na^+^ ratio is essential for many species to maintain a low concentration of Na^+^ [48, 49]. In this study, we investigated the salt tolerance of *TCP9-like*-overexpressing soybean plants by the root hydroponic assay with 150–250 mmol/L NaCl for 15 days. The overexpression of *TCP9-like* caused significantly lower accumulation of Na^+^ and higher accumulation of K^+^ than the WT plants exposed to 200 and 250 mmol/L NaCl. Accordingly, the K^+^/Na^+^ ratio of the transgenic plants was significantly higher than that of WT plants exposed to 200 and 250 mmol/L NaCl (**Fig 4**). The findings indicated that *TCP9-like* mediated the regulation of both Na^+^ and K^+^ accumulation in soybean, and contributed to the improved tolerance of soybean to salt stress.

*NHX* (vacuolar Na^+^/H^+^ antiporter), *SOS* (salt overly sensitive), and *HKT* (high-affinity K^+^ transporters) play critical roles in plant response to high salt stress [50–54]. NHX1 localized in the tonoplast could sequester the absorbed salt ions (especially Na^+^) into the vacuole to prevent the excess ion accumulation in the plant [55–57]. The overexpression of *CcSOS1* reduced the accumulation of Na^+^ and maintained a favorable K^+^/Na^+^ ratio compared with the WT plants [58]. The HKT1 transporter can also prevent the excess Na^+^ accumulation in the plant roots under salt stress [59, 60]. In this study, the overexpression of *TCP9-like* up-regulated the expression levels of *GmNHX1*, *GmNHX3*, *GmSOS1*, *GmSOS2-like*, and *GmHKT1* (**Fig 5**). Taken together, we hypothesized that *TCP9-like* may function as a positive regulator in the response to salt stress by regulating the expression of vacuolar K^+^/Na^+^ transporters and the critical genes in downstream biological pathways.

In conclusion, the expression of *TCP9-like* was induced under salt stress, and TCP9-like directly or indirectly regulated the expression of vacuolar K^+^/Na^+^ transporters (*GmNHX1* and *GmNHX3*) and the critical genes (*GmSOS1*, *GmSOS2-like*, and *GmHKT1*) in downstream pathways, leading to a significantly higher K^+^/Na^+^ ratio. Accordingly, the salt tolerance of *TCP9-like*-overexpressing soybean plants was improved.

## Supporting information

S1 Table Primer sequences used in the present study. (XLSX)

S2 Table Source data underlying the graphs presented in the main figures. (XLSX)

S1 Fig Nucleotide sequence and amino acid sequences of soybean TCP9-like gene. TCP domain was marked with a underlined. Sequence The sequence of TCP9-like gene was downloaded from the Phytozome database (http://www.phytozome.net/). TCP domain was identified from the InterPro software (http://www.ebi.ac.uk/interpro/scan.html/).
(TIF)

S2 Fig Detection of the T0 transgenic plants with PAT/Bar LibertyLink strips. WT, wild type. Labels 1–3, individual transgenic plants. The bands at red arrowhead indicate that BAR is positive. M, DL2000 DNA marker.
(TIF)

S3 Fig Detection of the T1 transgenic plants with PAT/Bar LibertyLink strips. WT, wild type. Labels 1–13/10/12, individual transgenic plants. The bands at red arrowhead indicate that BAR is positive. M, DL2000 DNA marker.
(TIF)

S4 Fig Detection of the T2 transgenic plants with PAT/Bar LibertyLink strips. WT, wild type. Labels 1–27/23/24, individual transgenic plants. The bands at red arrowhead indicate that BAR is positive. M, DL2000 DNA marker.
(TIF)

S1 Raw images (ZIP)
