# Supplementary figures and images for: Overexpression of TCP9-like gene enhances salt tolerance in transgenic soybean
Source: PLoS One. 2023 Jul 26;18(7):e0288985. doi: 10.1371/journal.pone.0288985 (PMC10370689; doi:10.1371/journal.pone.0288985)

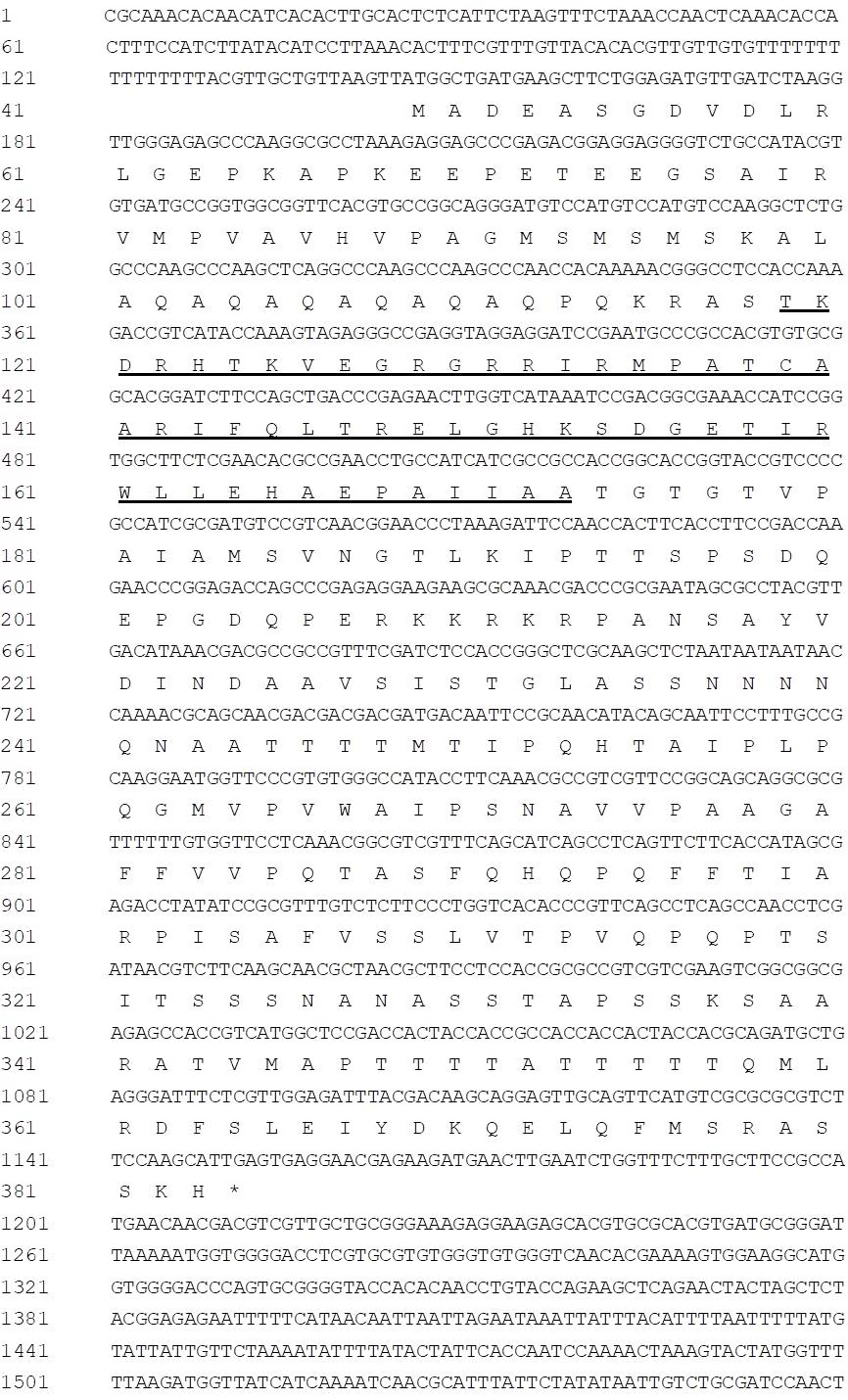

Supplement: S1 Fig — TCP domain was marked with a underlined. Sequence The sequence of TCP9-like gene was downloaded from the Phytozome database (http://www.phytozome.net/). TCP domain was identified from the InterPro software (http://www.ebi.ac.uk/interpro/scan.html/). (TIF) [file pone.0288985.s003.tif]

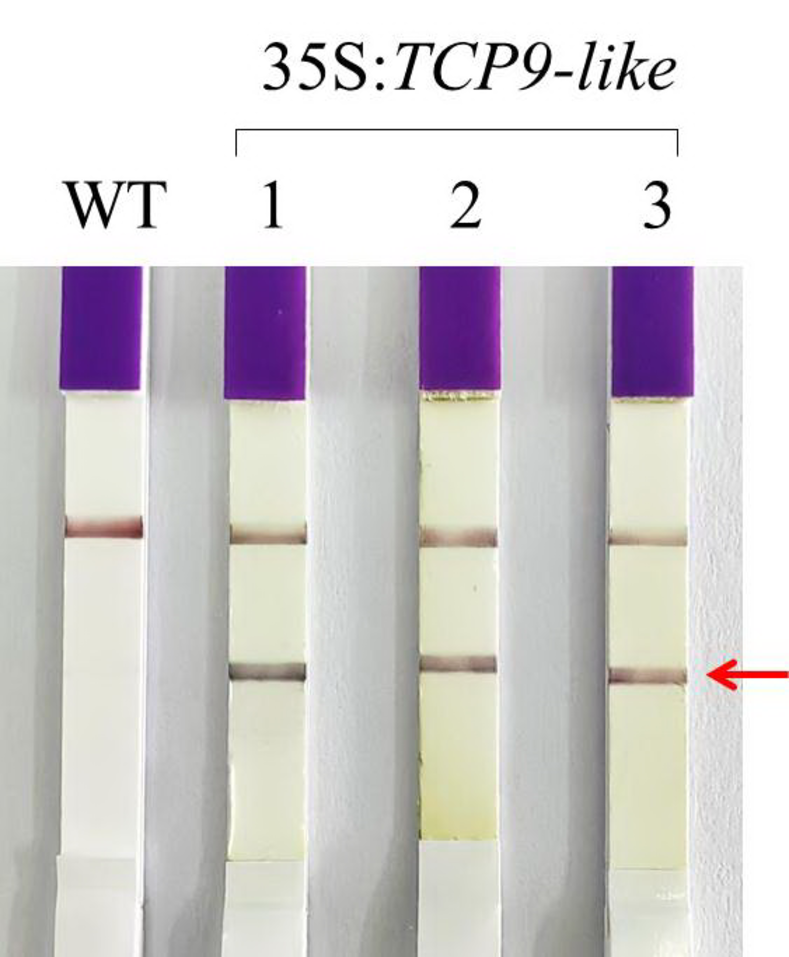

Supplement: S2 Fig — WT, wild type. Labels 1–3, individual transgenic plants. The bands at red arrowhead indicate that BAR is positive. M, DL2000 DNA marker. (TIF) [file pone.0288985.s004.tif]

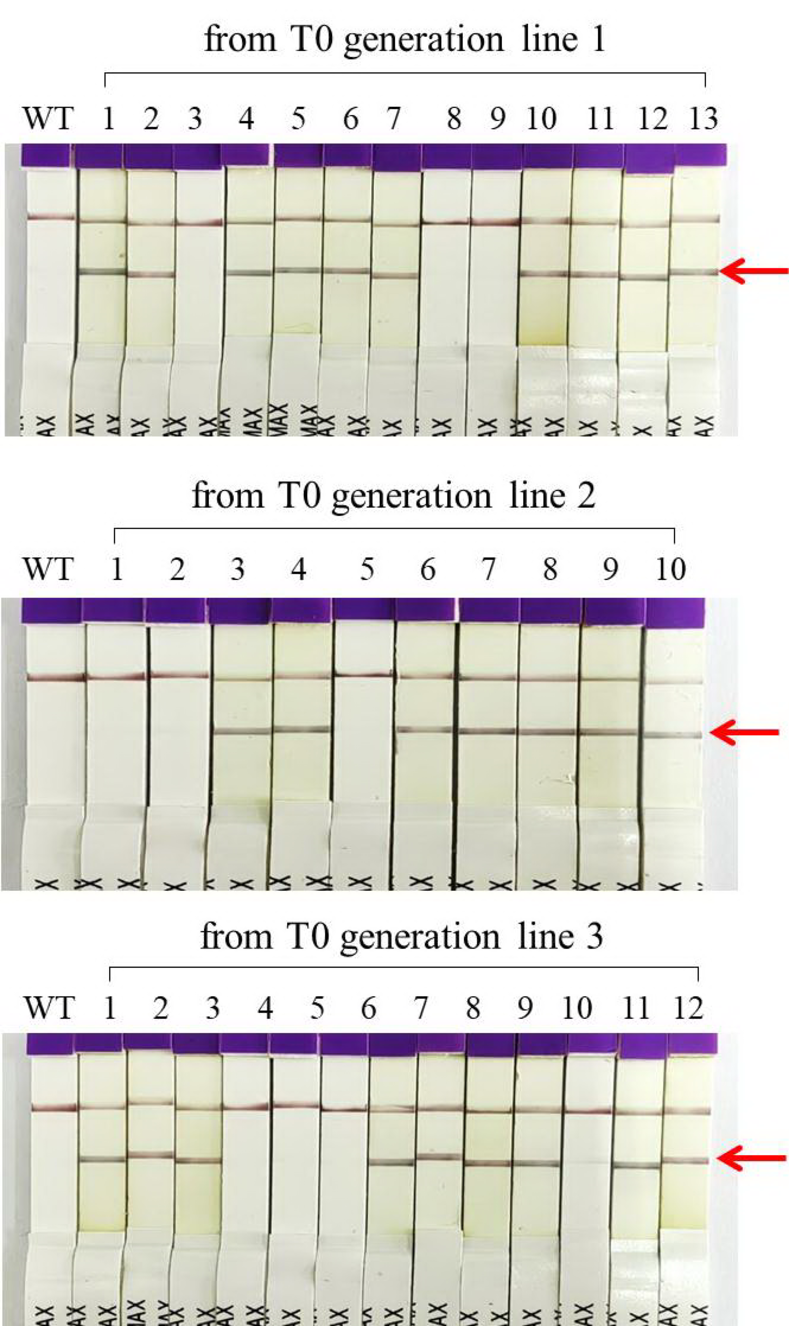

Supplement: S3 Fig — WT, wild type. Labels 1–13/10/12, individual transgenic plants. The bands at red arrowhead indicate that BAR is positive. M, DL2000 DNA marker. (TIF) [file pone.0288985.s005.tif]

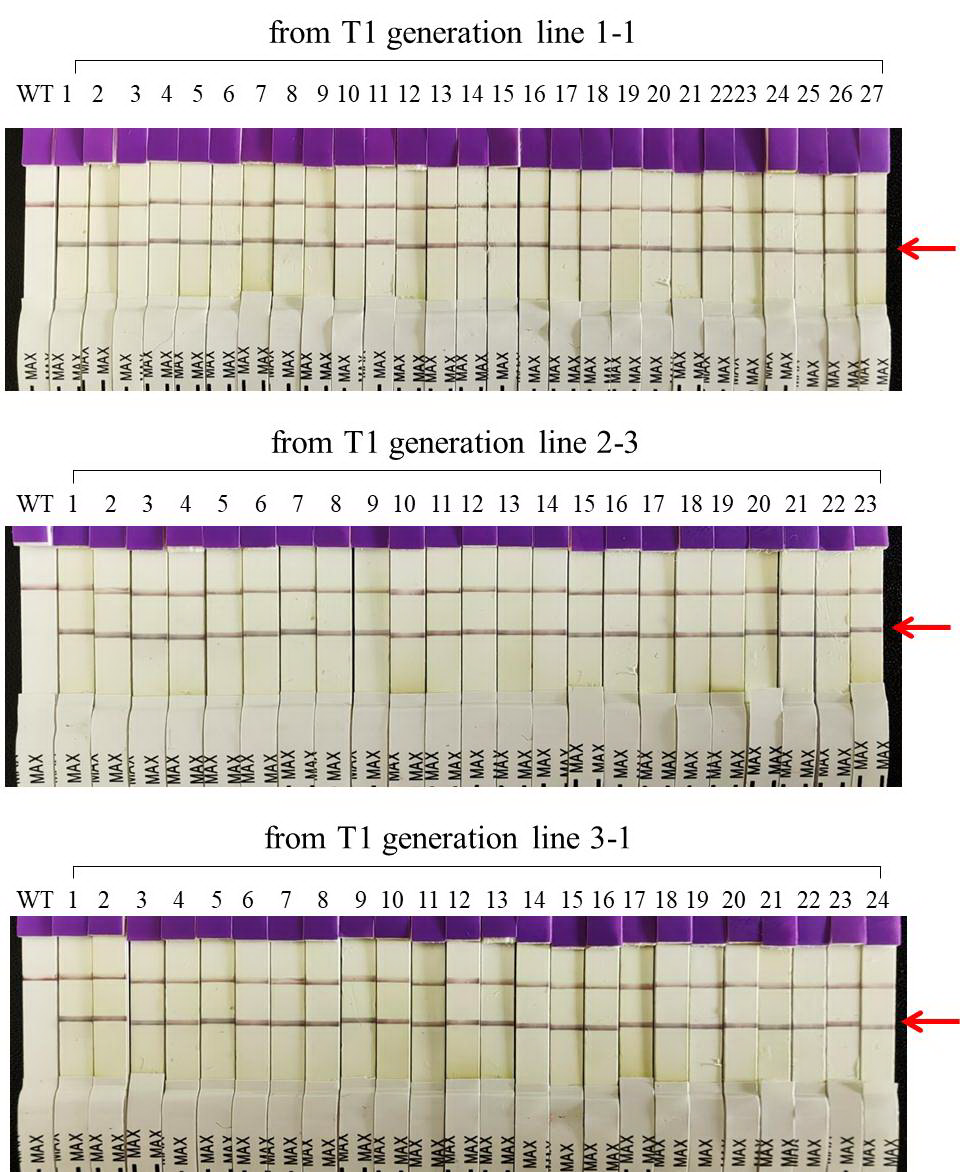

Supplement: S4 Fig — WT, wild type. Labels 1–27/23/24, individual transgenic plants. The bands at red arrowhead indicate that BAR is positive. M, DL2000 DNA marker. (TIF) [file pone.0288985.s006.tif]

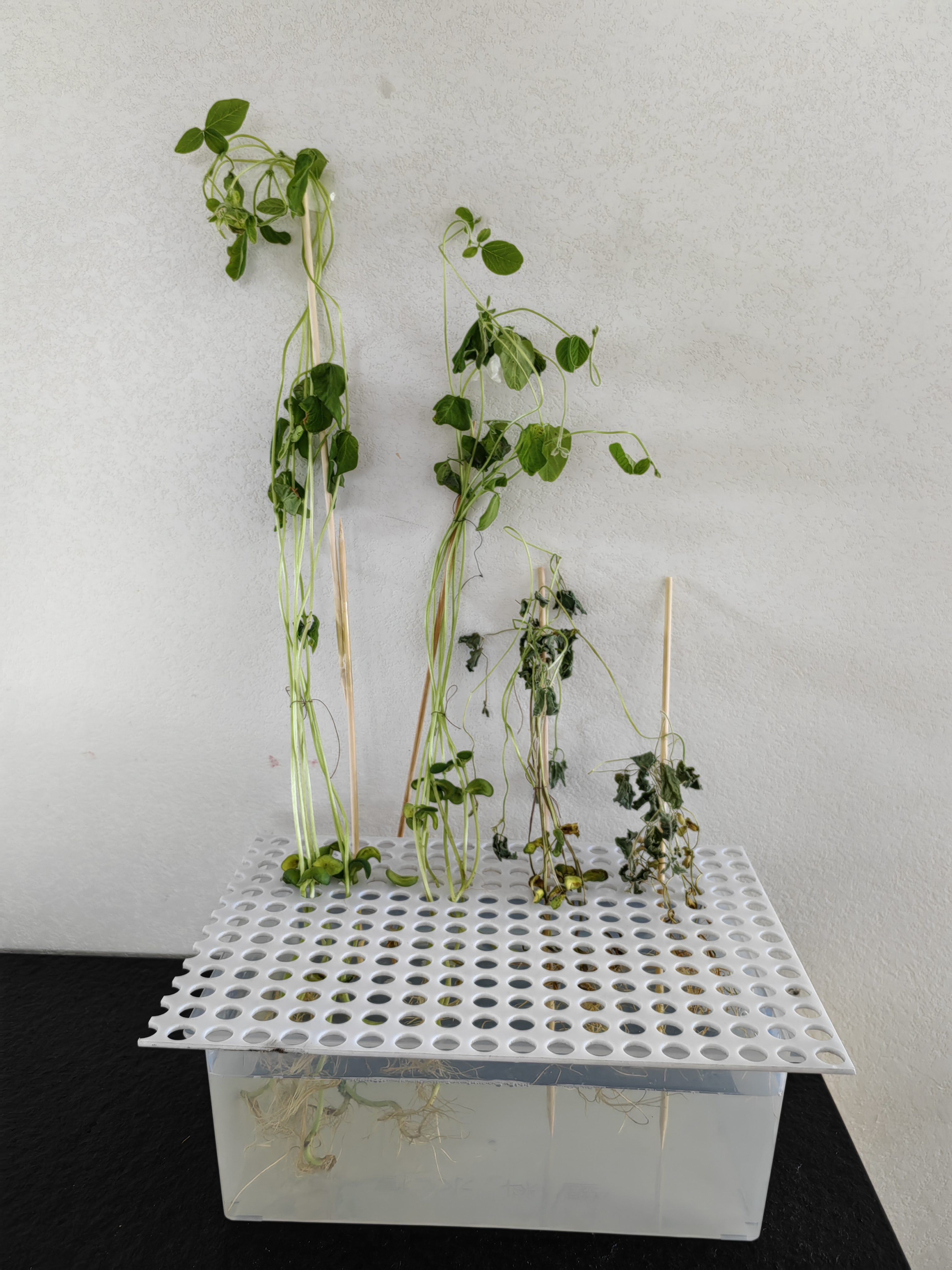

Supplement: S1 Raw images — (ZIP) [file pone.0288985.s007.zip › Fig 2A.jpg]

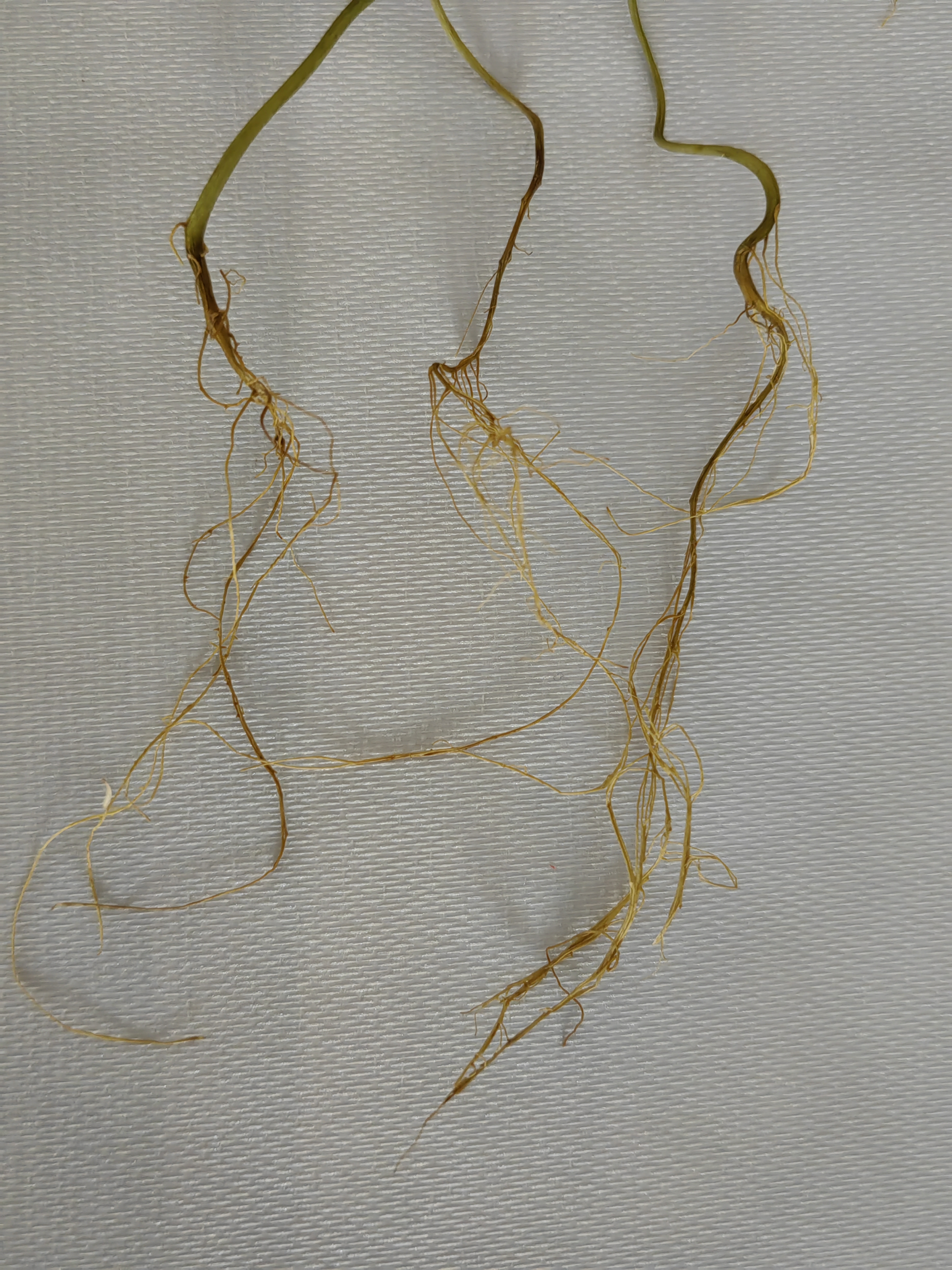

Supplement: S1 Raw images — (ZIP) [file pone.0288985.s007.zip › Fig 2B DN50.jpg]

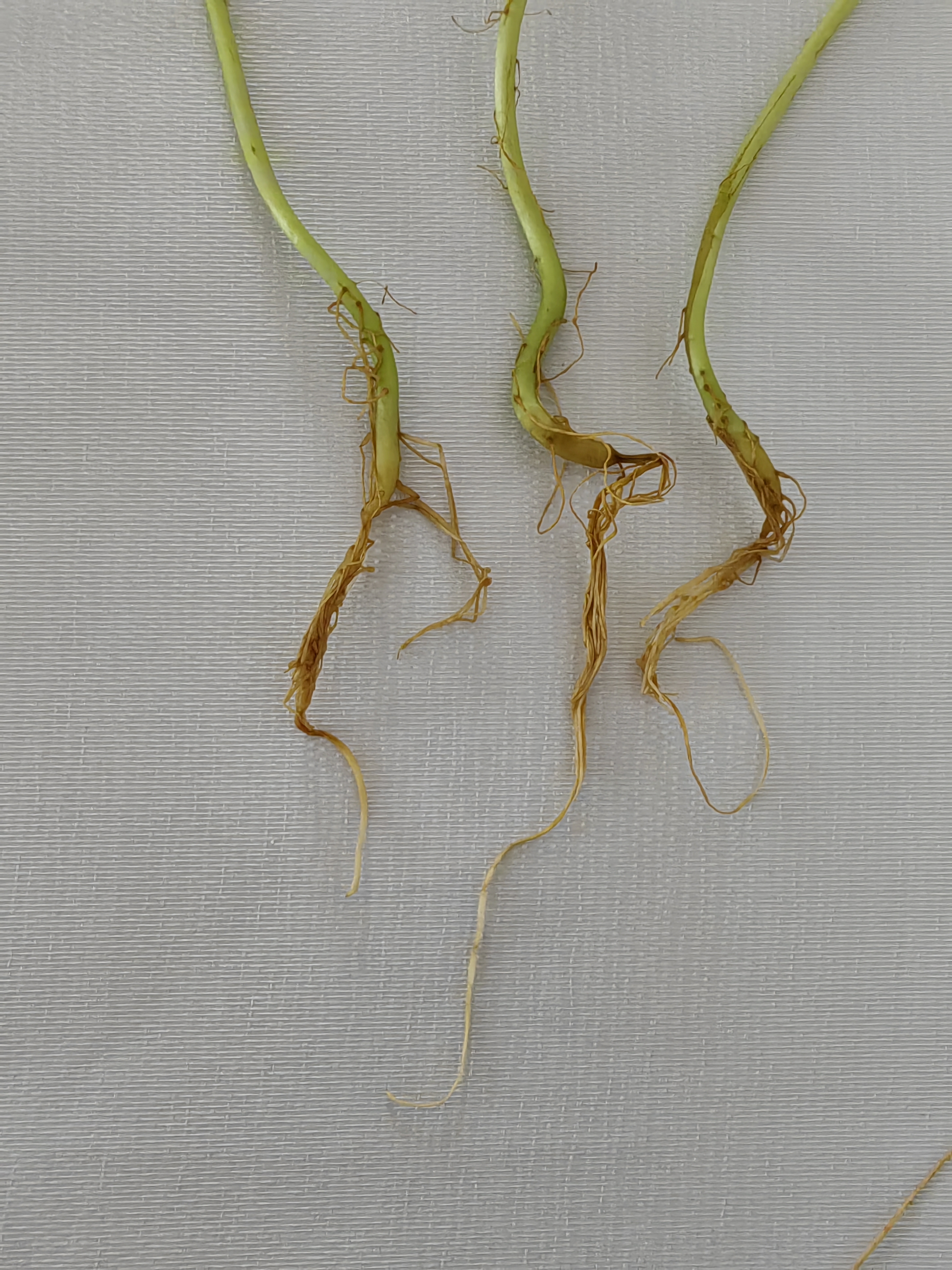

Supplement: S1 Raw images — (ZIP) [file pone.0288985.s007.zip › Fig 2B JN18-2.jpg]

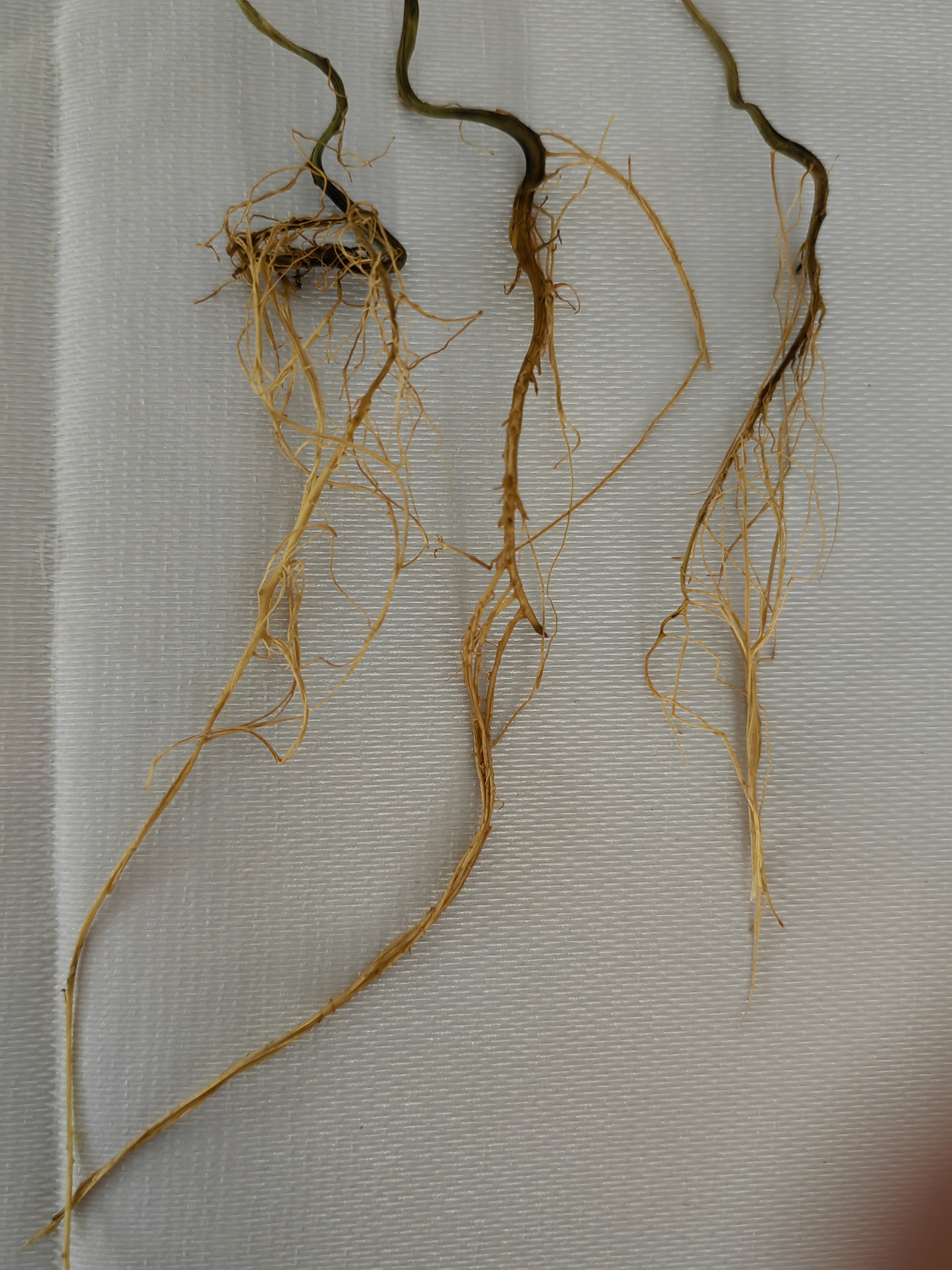

Supplement: S1 Raw images — (ZIP) [file pone.0288985.s007.zip › Fig 2B JN18-7.jpg]

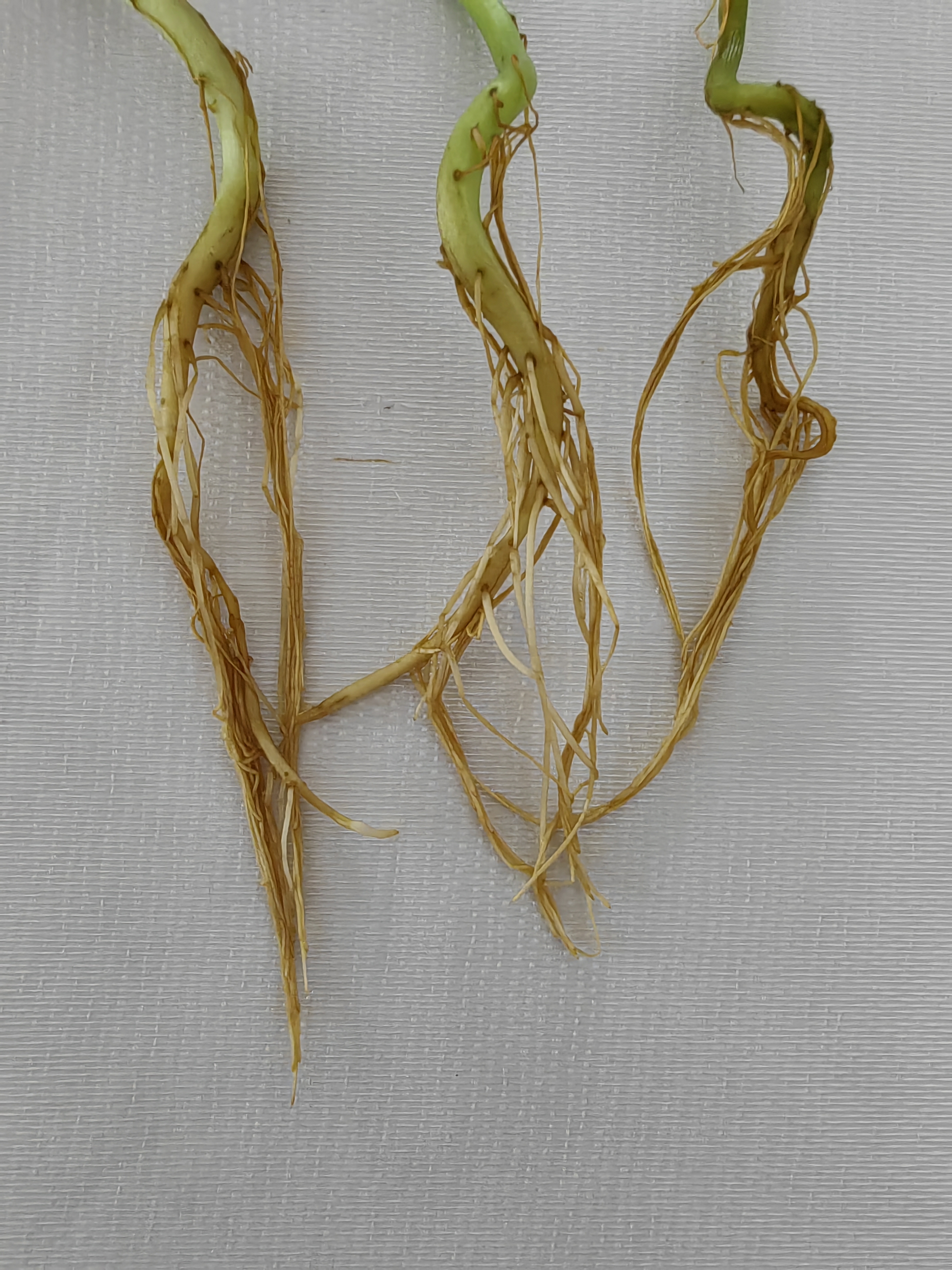

Supplement: S1 Raw images — (ZIP) [file pone.0288985.s007.zip › Fig 2B JN30.jpg]

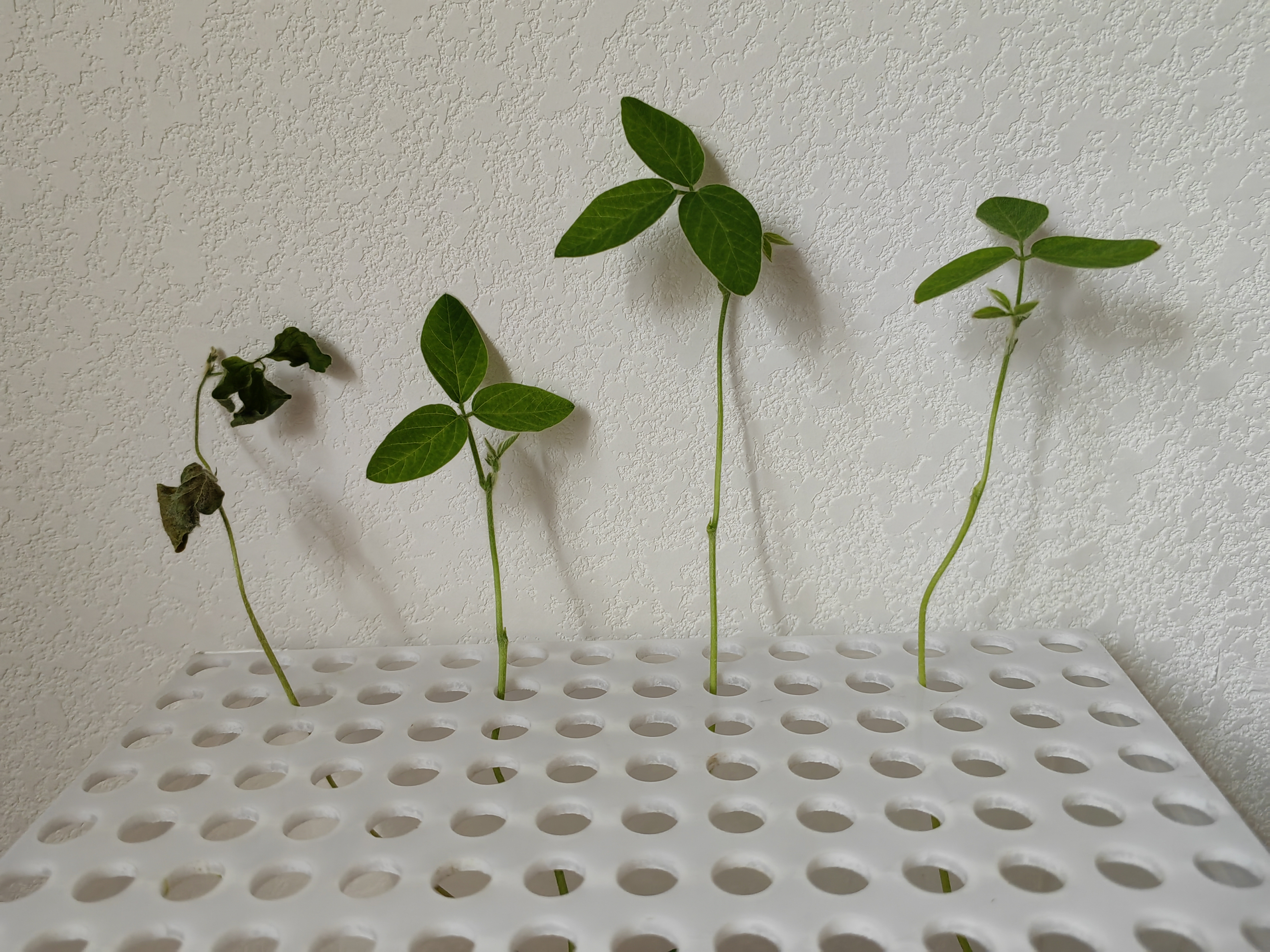

Supplement: S1 Raw images — (ZIP) [file pone.0288985.s007.zip › Fig 3A-1.jpg]

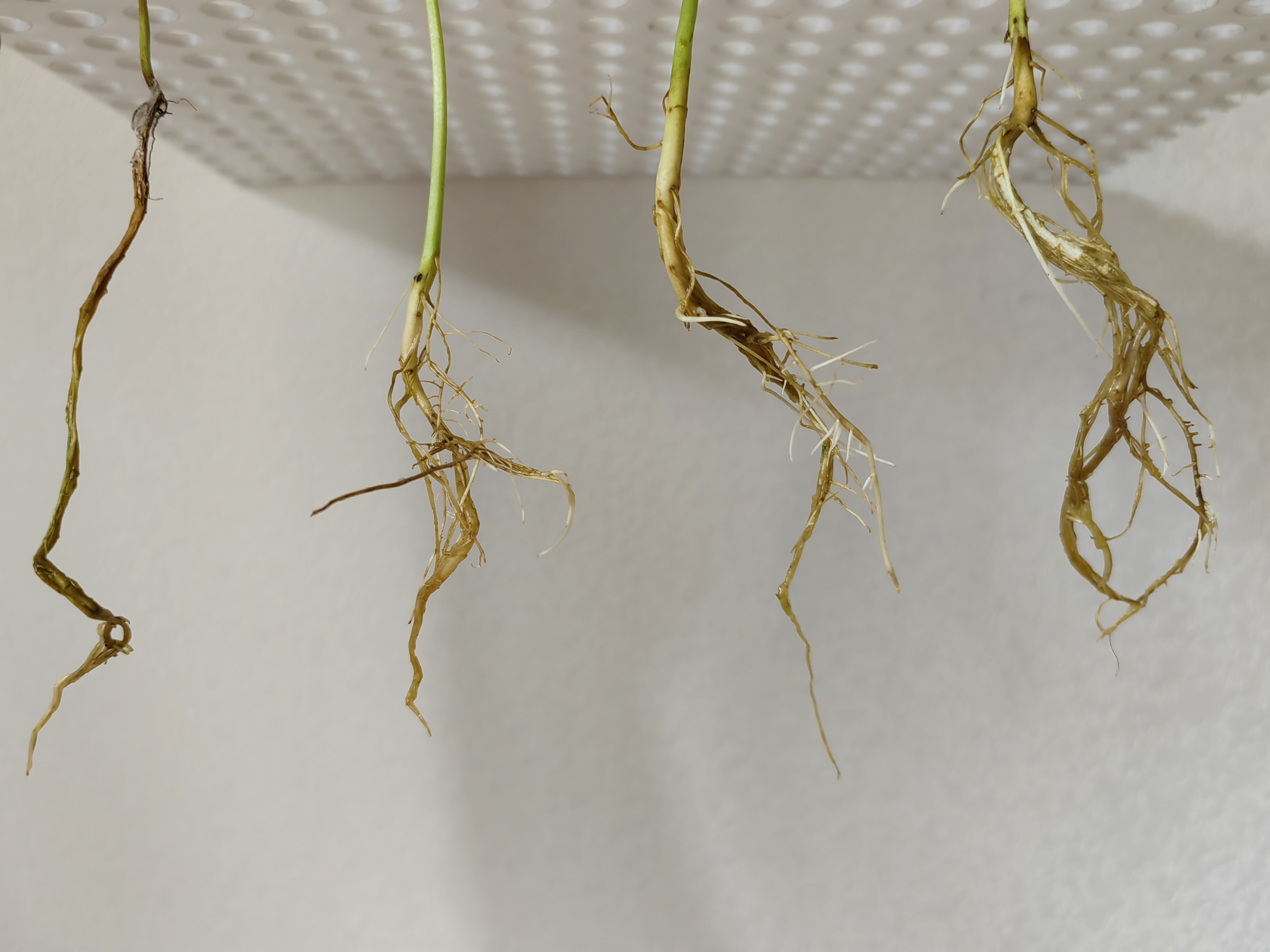

Supplement: S1 Raw images — (ZIP) [file pone.0288985.s007.zip › Fig 3A-2.jpg]

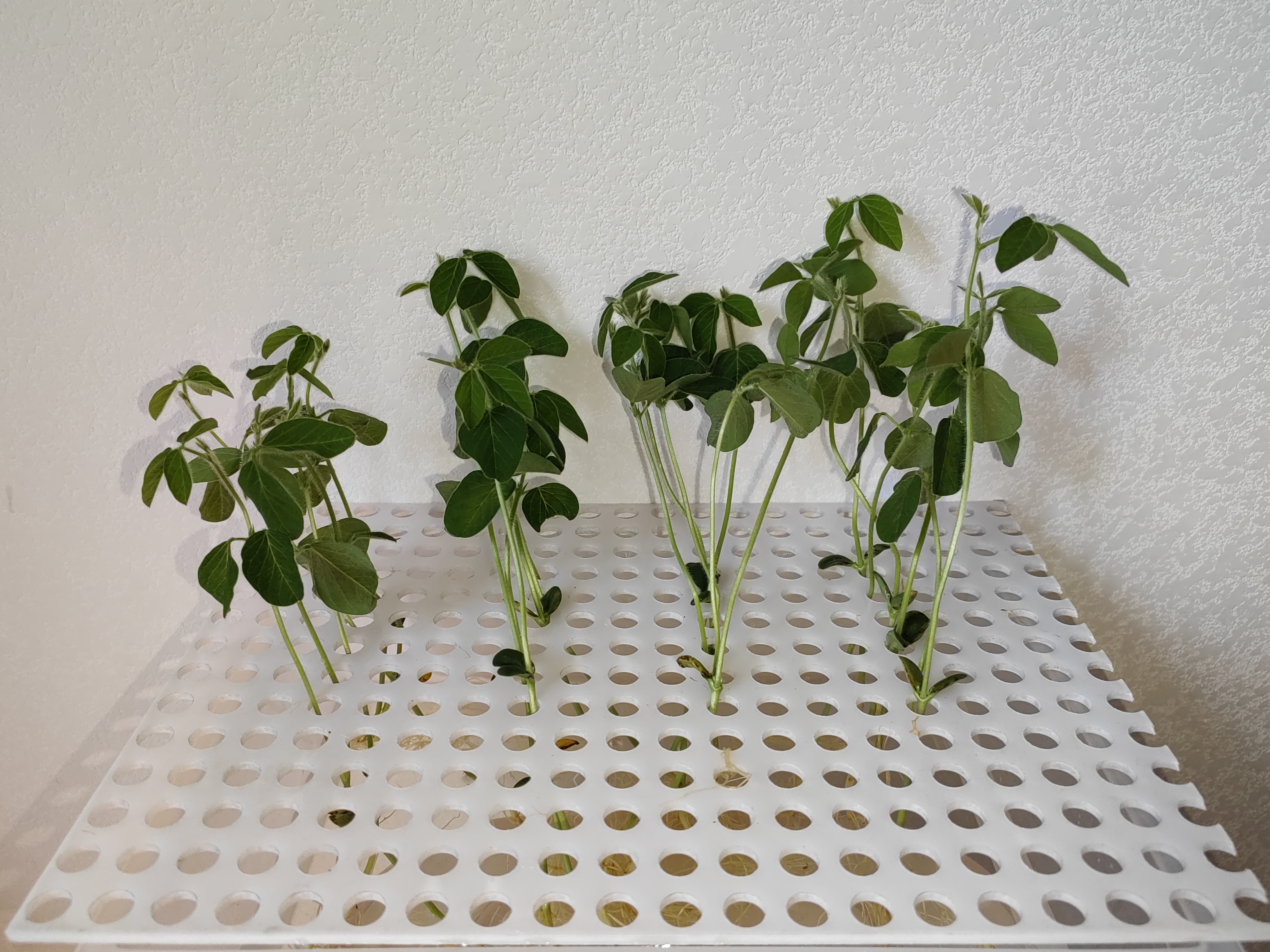

Supplement: S1 Raw images — (ZIP) [file pone.0288985.s007.zip › Fig 4A 150mM.jpg]

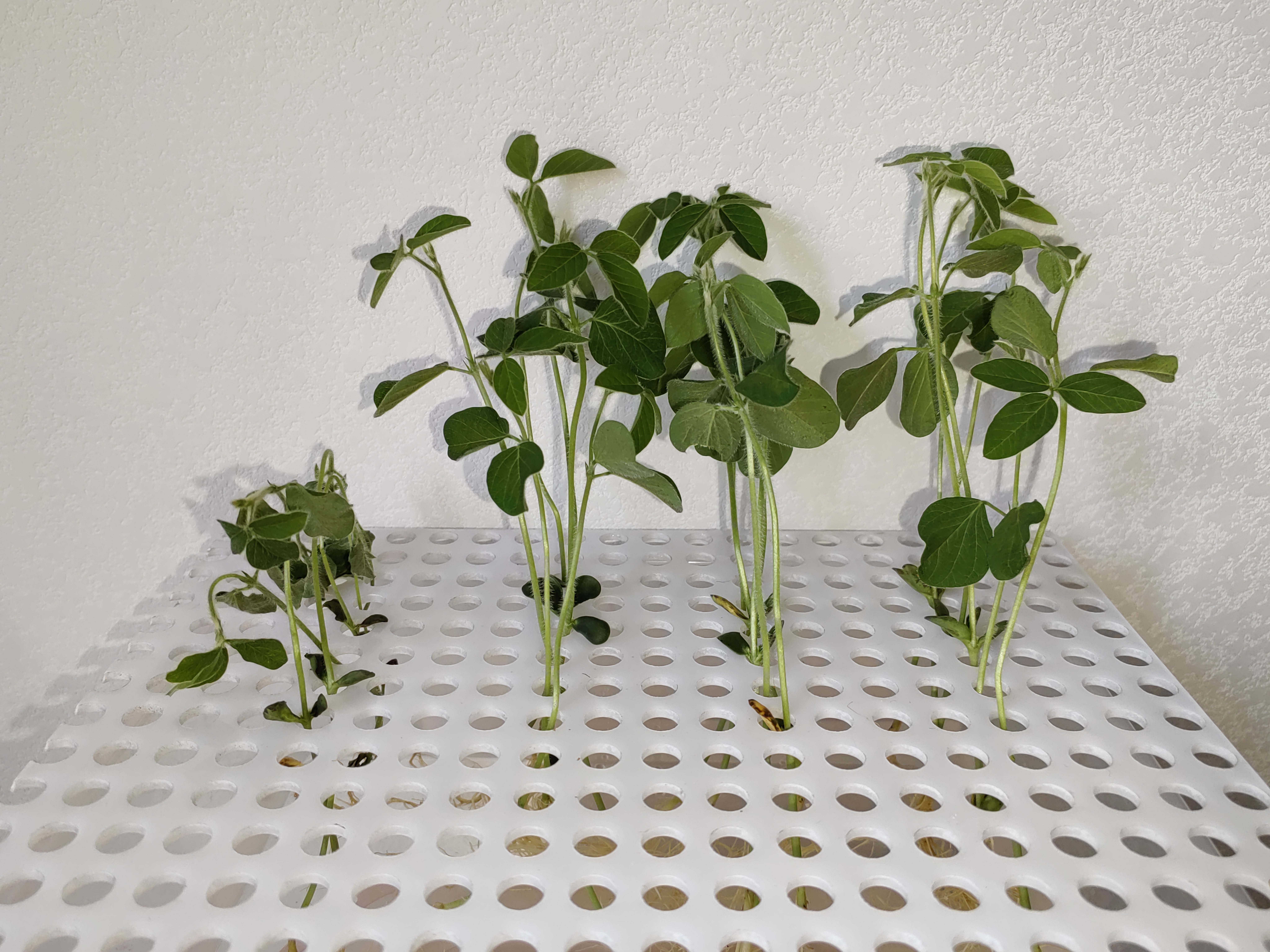

Supplement: S1 Raw images — (ZIP) [file pone.0288985.s007.zip › Fig 4A 200mM.jpg]

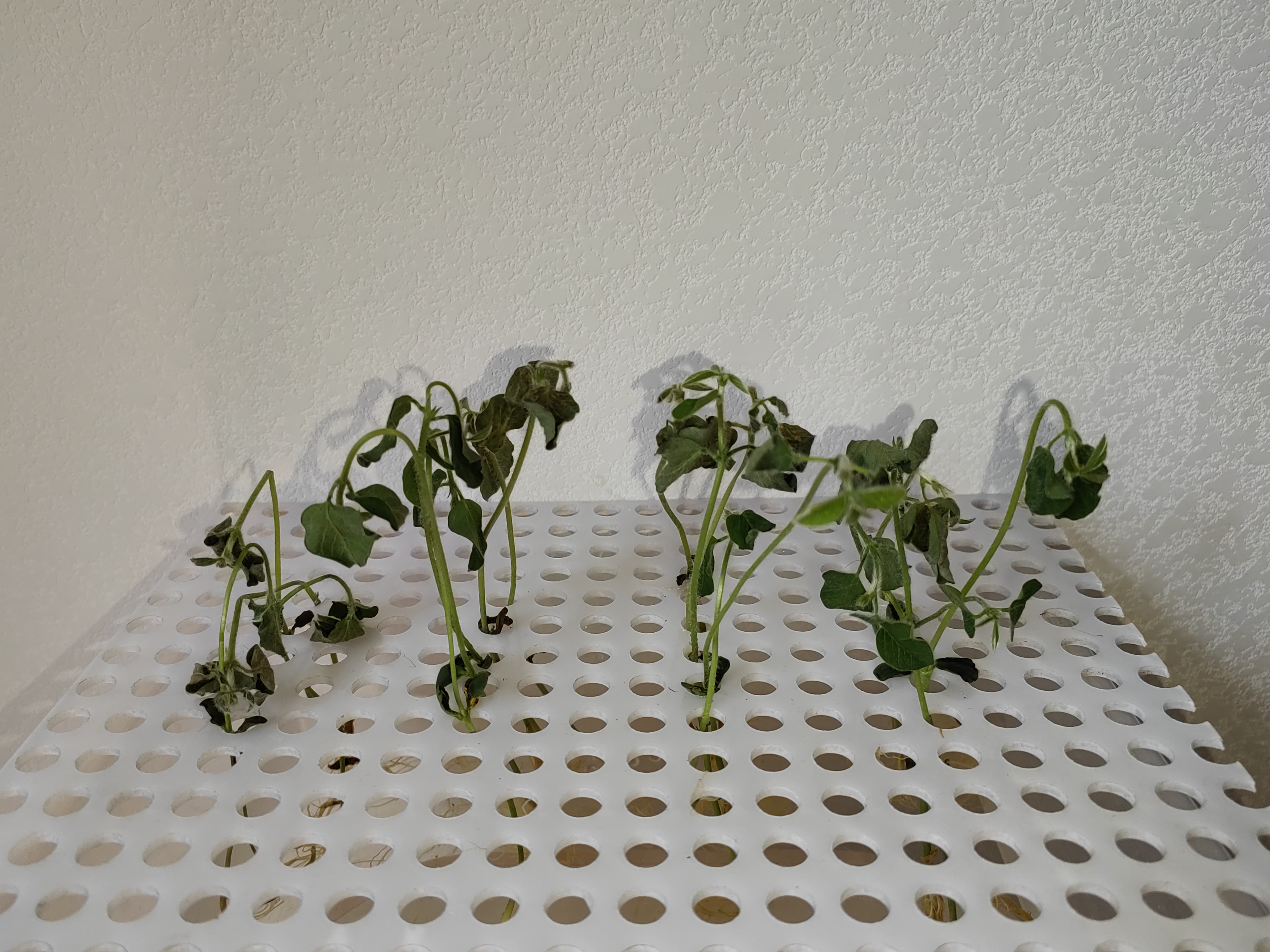

Supplement: S1 Raw images — (ZIP) [file pone.0288985.s007.zip › Fig 4A 250mM.jpg]

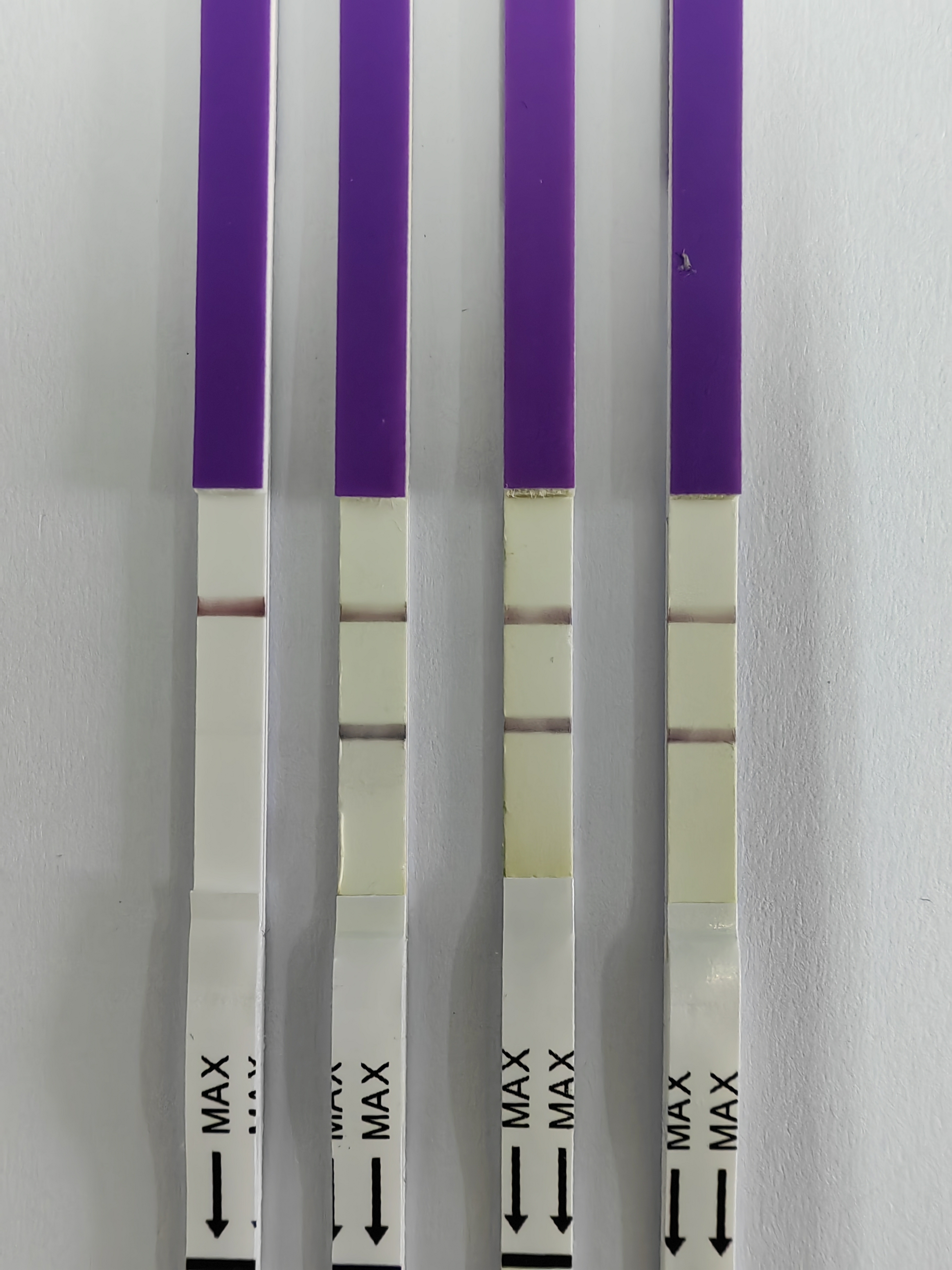

Supplement: S1 Raw images — (ZIP) [file pone.0288985.s007.zip › Fig S2.jpg]

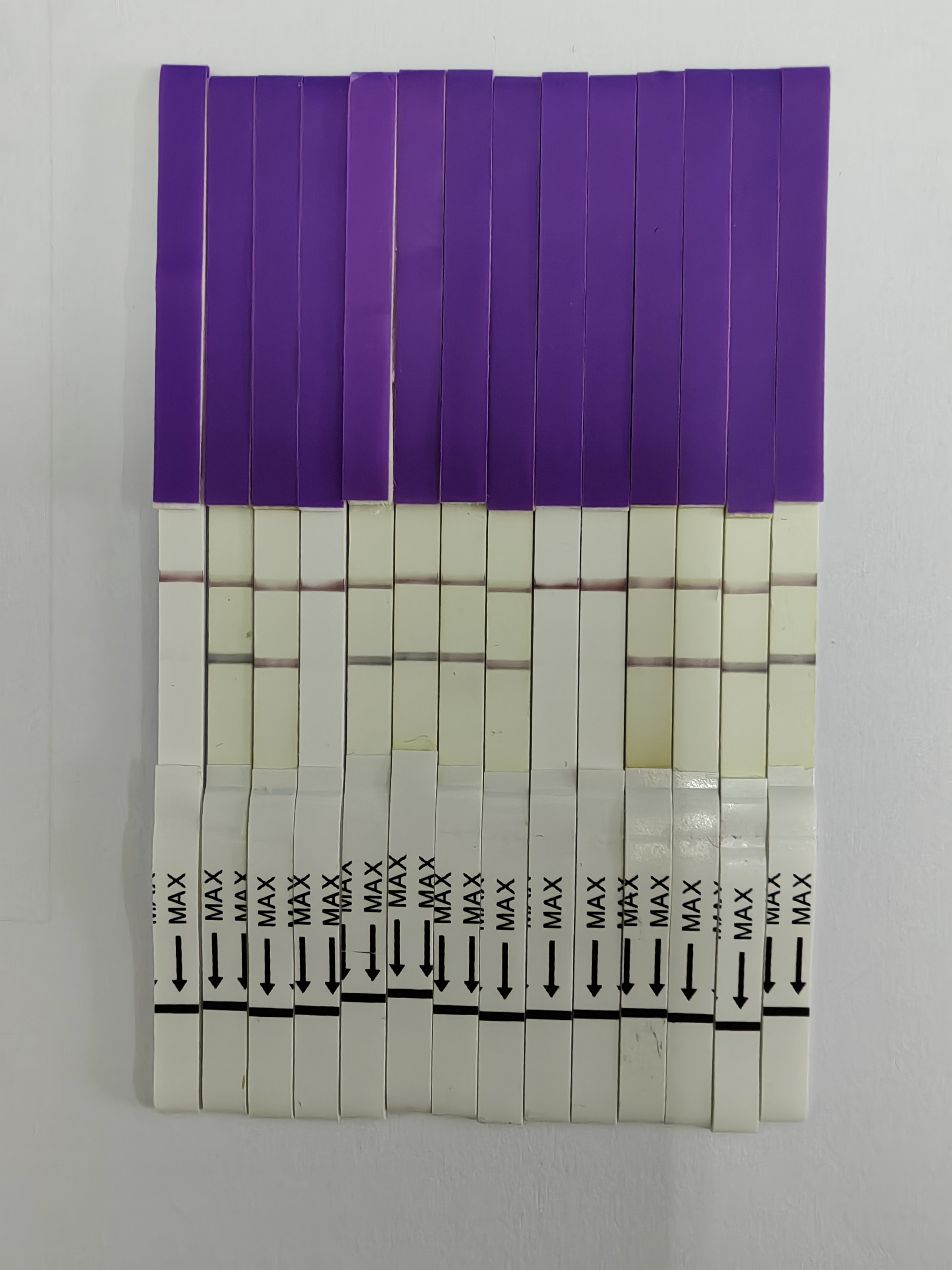

Supplement: S1 Raw images — (ZIP) [file pone.0288985.s007.zip › Fig S3 line 1.jpg]

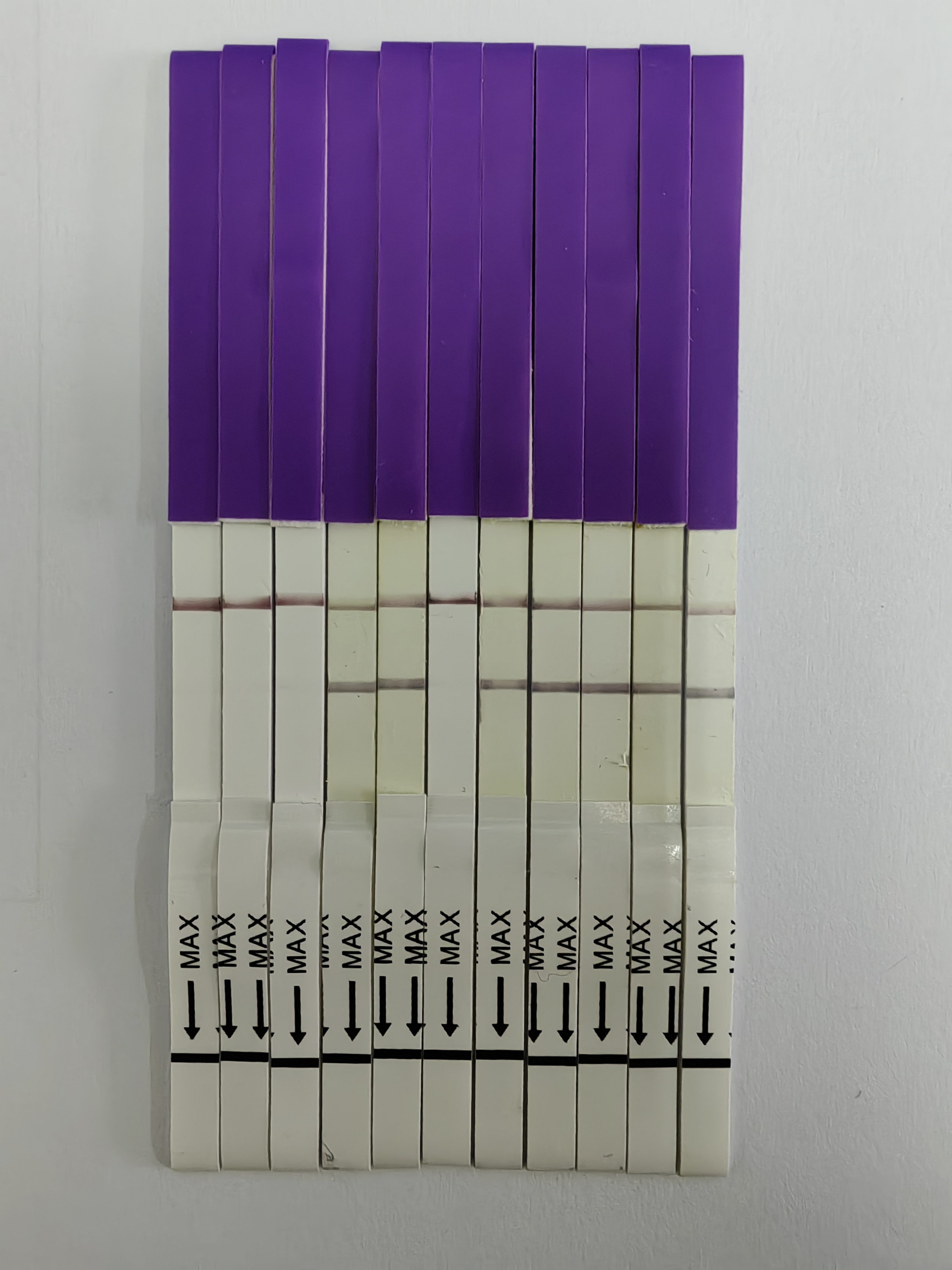

Supplement: S1 Raw images — (ZIP) [file pone.0288985.s007.zip › Fig S3 line 2.jpg]

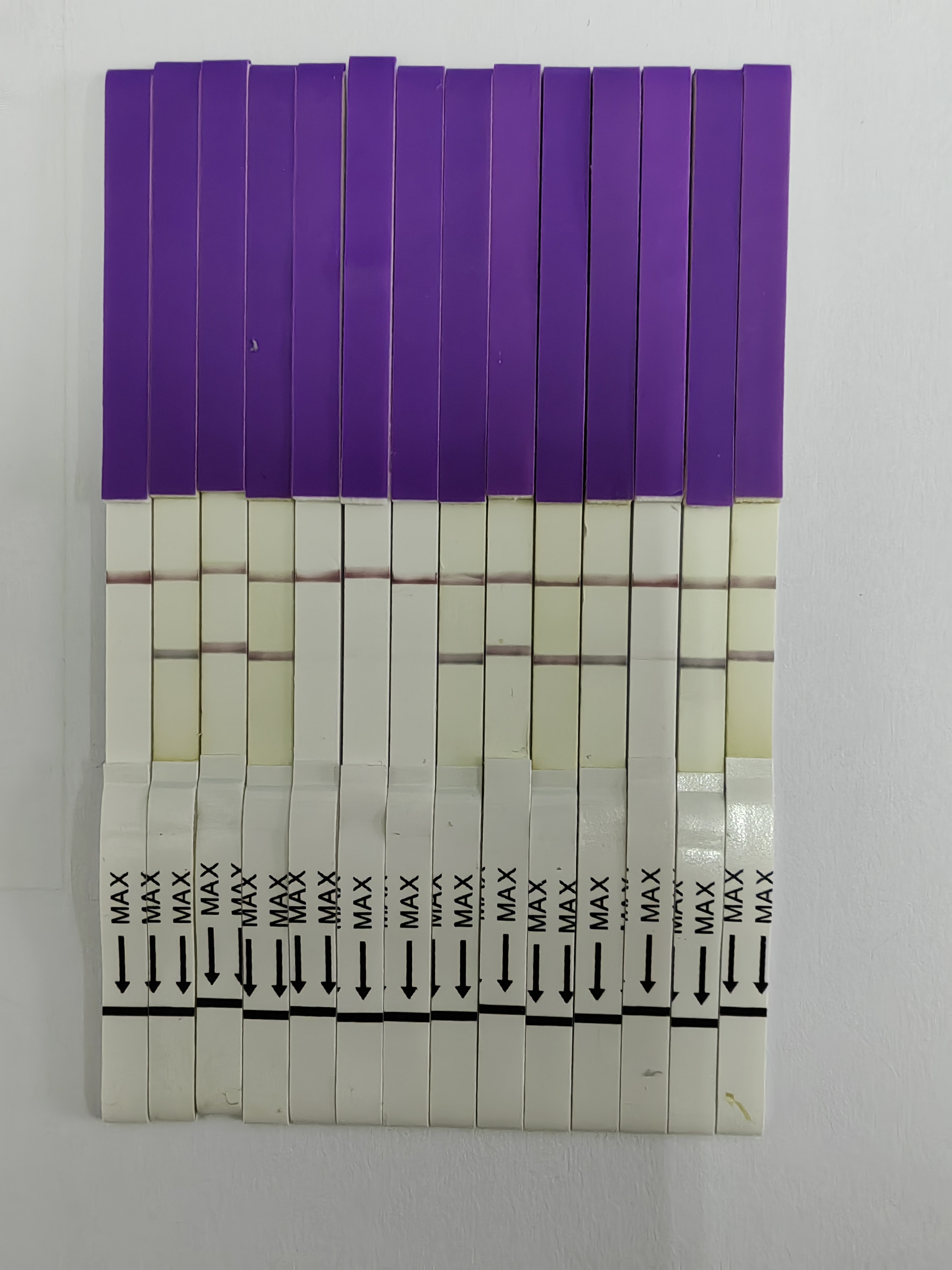

Supplement: S1 Raw images — (ZIP) [file pone.0288985.s007.zip › Fig S3 line 3.jpg]

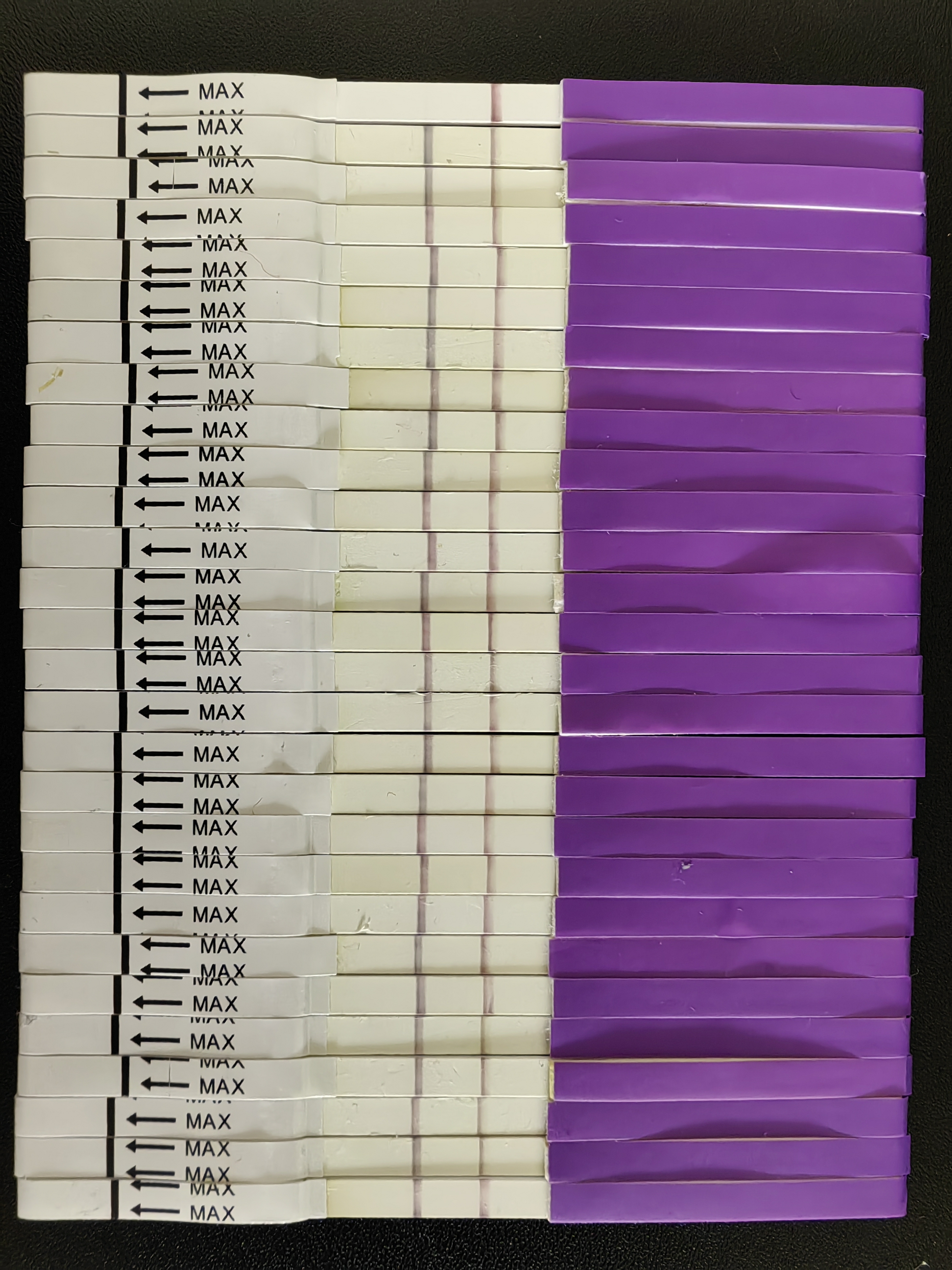

Supplement: S1 Raw images — (ZIP) [file pone.0288985.s007.zip › Fig S4 line 1-1.jpg]

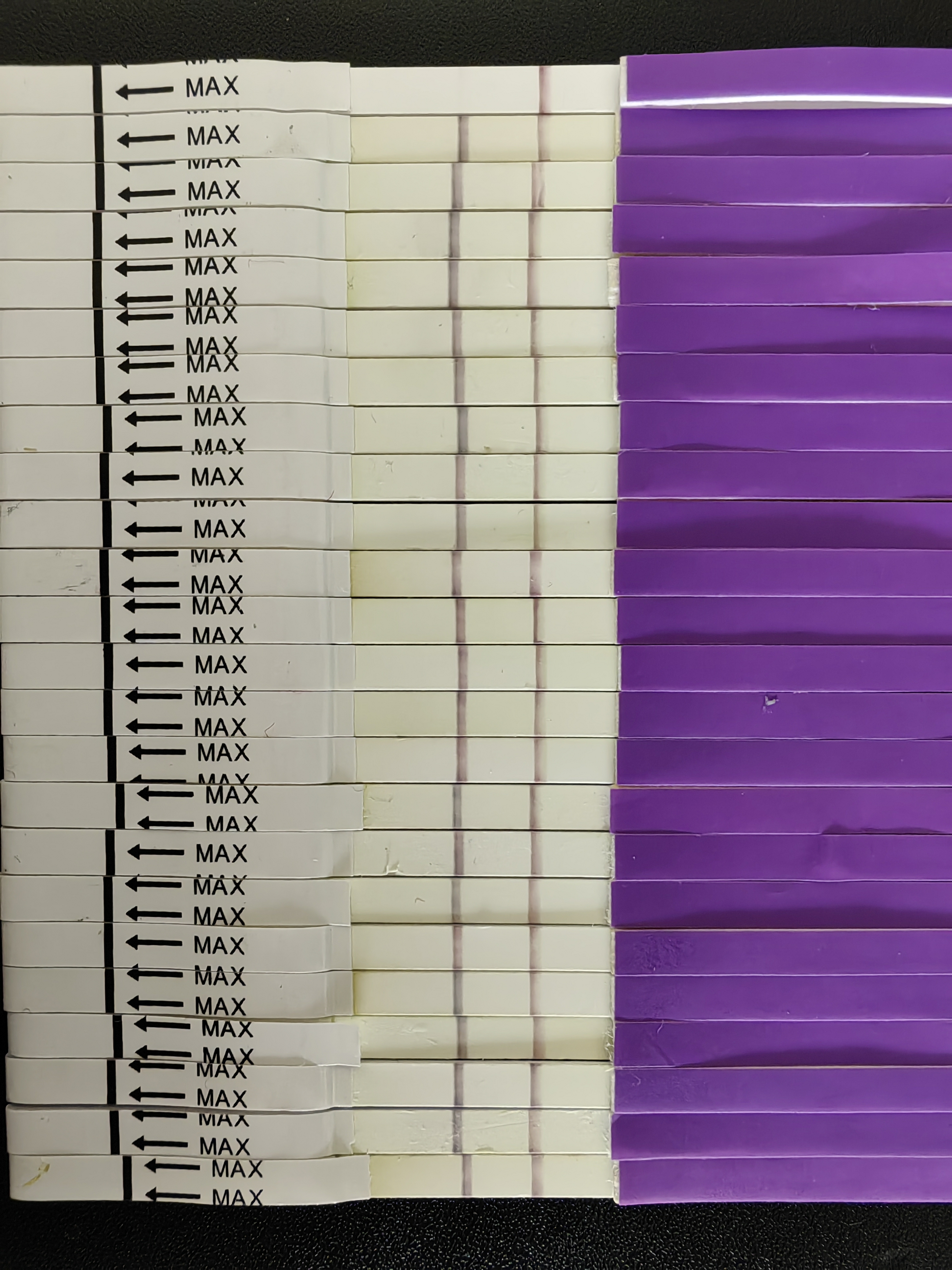

Supplement: S1 Raw images — (ZIP) [file pone.0288985.s007.zip › Fig S4 line 2-3.jpg]

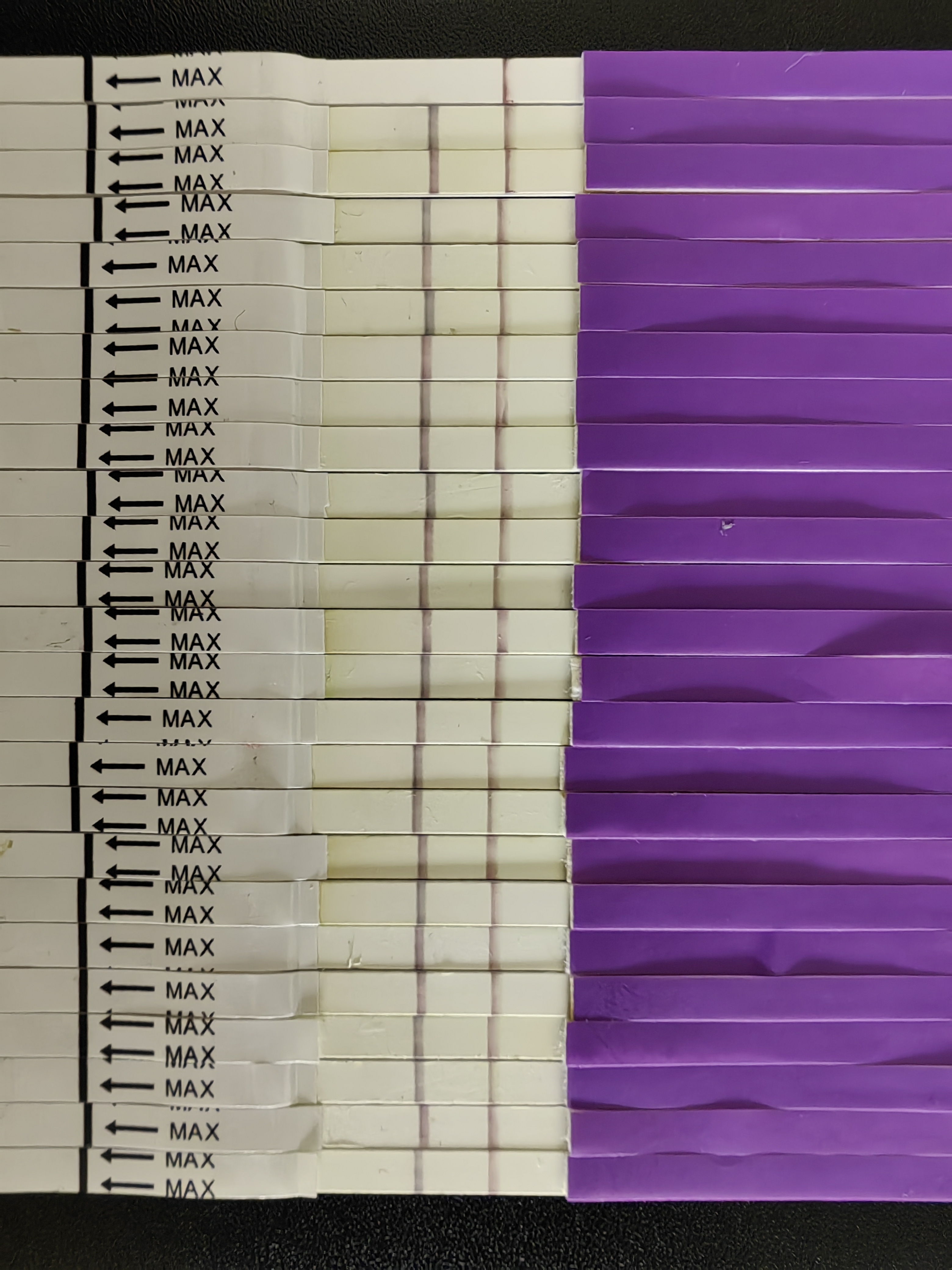

Supplement: S1 Raw images — (ZIP) [file pone.0288985.s007.zip › Fig S4 line 3-1.jpg]
